# Genome-wide analysis of ATP-binding cassette (ABC) transporters in the sweetpotato whitefly, *Bemisia tabaci*

**DOI:** 10.1186/s12864-017-3706-6

**Published:** 2017-04-26

**Authors:** Lixia Tian, Tianxue Song, Rongjun He, Yang Zeng, Wen Xie, Qingjun Wu, Shaoli Wang, Xuguo Zhou, Youjun Zhang

**Affiliations:** 1 Department of Plant Protection, Institute of Vegetables and Flowers, Chinese Academy of Agricultural Sciences, Beijing, 100081 China; 2 College of Life Science, Northeast Agricultural University, Harbin, 150030 China; 3 Department of Entomology, University of Kentucky, Lexington, KY 40546-0091 USA

**Keywords:** ATP-binding cassette transporter, *Bemisia tabaci*, Genome, Gene family expansion, Invasiveness

## Abstract

**Background:**

ABC transporter superfamily is one of the largest and ubiquitous groups of proteins. Because of their role in detoxification, insect ABC transporters have gained more attention in recent years. In this study, we annotated ABC transporters from a newly sequenced sweetpotato whitefly genome. *Bemisia tabaci* Q biotype is an emerging global invasive species that has caused extensive damages to field crops as well as ornamental plants.

**Results:**

A total of 55 ABC transporters containing all eight described subfamilies (A to H) were identified in the *B. tabaci* Q genome, including 8 ABCAs, 3 ABCBs, 6 ABCCs, 2 ABCDs, 1 ABCE, 3 ABCFs, 23 ABCGs and 9 ABCHs. In comparison to other species, subfamilies G and H in both phloem- and blood-sucking arthropods are expanded. The temporal expression profiles of these 55 ABC transporters throughout *B. tabaci* developmental stages and their responses to imidacloprid, a neonicotinoid insecticide, were investigated using RNA-seq analysis. Furthermore, the mRNA expression of 24 ABC transporters (44% of the total) representing all eight subfamilies was confirmed by the quantitative real-time PCR (RT-qPCR). Furthermore, mRNA expression levels estimated by RT-qPCR and RNA-seq analyses were significantly correlated (*r* = 0.684, *p* < 0.01).

**Conclusions:**

It is the first genome-wide analysis of the entire repertoire of ABC transporters in *B. tabaci*. The identification of these ABC transporters, their temporal expression profiles during *B. tabaci* development, and their response to a neonicotinoid insecticide lay the foundation for functional genomic understanding of their contribution to the invasiveness of *B. tabaci.*

**Electronic supplementary material:**

The online version of this article (doi:10.1186/s12864-017-3706-6) contains supplementary material, which is available to authorized users.

## Background

ATP-binding cassette (ABC) transporters constitute one of the largest protein superfamily and exists in all living organisms [1–3]. These transporters share highly conserved nucleotide binding domains (NBDs), which contain Walker A, Walker B and a signature motif, the C loop, linking the two Walker boxes [4, 5]. In addition to NBDs, a eukaryotic ABC transporter typically consists of one or two transmembrane domains (TMDs), which contain 6–11 membrane-spanning α-helices and dictate substrate specificity [1]. A classic domain architecture of a full-transporter has a TMD-NBD-TMD-NBD arrangement from the N- to C-terminus, while half-transporters have only one set of NBD-TMD. Based on the homology of their NBDs, human ABC transporters have been categorized into seven subfamilies, ABCA to ABCG [1]. ABCH, which was first discovered in *Drosophila melanogaster,* exists in arthropods and zebrafish but not in mammals, plants and fungi [6–12].

Most ABC transporters encodes membrane-bound proteins that carry a wide range of molecules (e.g., amino acids, peptides, vitamins, sugars, lipids, sterols, hormones, endogenous metabolites, inorganics and xenobiotics) across membranes [1]. ABC transporters use energy released by ATP hydrolysis at the NBDs to transport molecular cargoes across the membrane. ABC transporters also function in cell signaling, ribosome assembly and translation [3, 13]. For example, ABCE and ABCF proteins do not function as classic transporters but participate in transcription, translation and ribosome assembly [14, 15]. Furthermore, plant ABC transporters also contribute to osmolality and phytoalexin functions (plant-pathogen interactions) [16].

The advent of Genomics Era has greatly improved our understanding of the diversity and function of invertebrate ABC transporters, including 56 in the fruit fly *D. melanogaster* [1], 64 in the water flea *Daphnia pulex* [13], 51 in the silkworm *Bombyx mori* [8], 73 in the red flour beetle *Tribolium castaneum* [17] and 103 in the spider mites *Tetranychus urticae* [10]. Based on the transcriptome data, Epis *et al*. suggested that ABC transporters were involved in the permethrin resistance in a malaria vector, *Anopheles stephensi* [18]. Similarly, ABC transporters were implicated in the degradation of plant secondary metabolites in the cotton bollworm, *Helicoverpa armigera* [19]*.*

The sweetpotato whitefly, *Bemisia tabaci* (Gennadius) (Hemiptera: Aleyrodidae), is a phloem-feeding insect pest that cause substantial damages through feeding on over 600 host plants as well as by transmitting over 100 plant viruses [20]. As one of the most invasive pests, *B. tabaci* is generally considered as a species complex consisting of many biotypes or putative species with different biological and genetic characteristics [21]. The two most invasive and destructive *B. tabaci* biotypes are the Q (also known as Mediterranean, MED) and B (also known as Middle East-Asia Minor 1, MEAM1) [21]. *B. tabaci* has demonstrated a remarkable ability to develop resistance to insecticides in the field, including organophosphates, carbamates, pyrethroids, neonicotinoids and juvenile hormone mimics [22–24]. Insecticide was the driving force facilitating the *B. tabaci* biotype replacement events in China (B replaced indigenous biotypes in early 2000, and the subsequent replacement by Q in 2009) [25]. The overall goal of this research is to understand the involvement of ABC transporters, a family of phase III detoxification enzymes, in the metabolic resistance in *B. tabaci.* To archive this goal, we 1) provided the first genome-wide analysis of the entire repertoire of ABC transporters in the whitefly *B. tabaci* Q; 2) investigated the temporal expression profiles of these ABC transporters in different developmental stages; and 3) documented their transcriptomic response to imidacloprid, a neonicotinoid insecticide.

## Results and discussion

### Identification of ABC transporters in *B. tabaci*

**Fig. 1:**
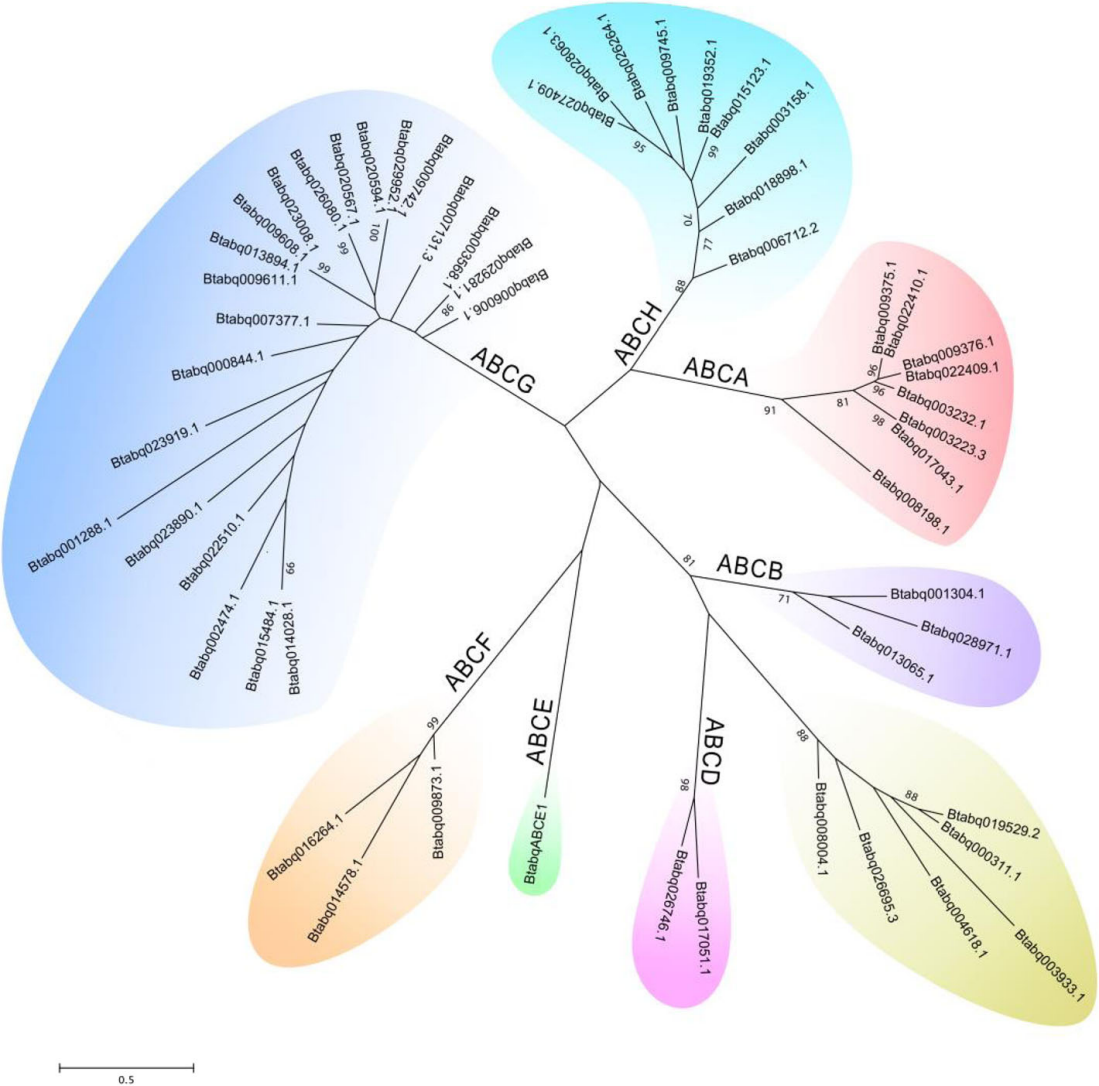
Phylogenetic relationship of 55 ABC transporters in *B. tabaci* Q. Amino acid sequences of nucleotide-binding domains (NBDs) were aligned using ClustalW and subjected to a maximum likelihood analysis by MEGA5 [97]. Numbers at the branch point of the node represent the value resulting from 1000 replications. All positions with less than 95% site coverage were eliminated

A total of 55 ABC transporters was identified in *B. tabaci* Q genome (Table 1). The number of ABC transporters in *B. tabaci* Q is comparable with other arthropod species; however, subfamilies G and H expanded in phloem- and blood-sucking arthropods (Table 2 and Additional file 1: Figure S1). For example, ABCG and ABCH account for 58% (42 and 16%, respectively) of ABC transporters in the phloem-sucking *B. tabaci*, and 49% (45 and 4%, respectively) in the blood-sucking *Cimex lectularius*. The percentage reduces to 21% (18% + 3%) in the red flour beetle, *T. castaneum.* The expansion of ABCG and ABCH subfamilies in these polyphagous and phloem-sucking arthropods, including the whitefly *B. tabaci,* the wheat aphid *D. noxia* and the spider mite *T. urticae*, suggest the potential contribution of ABC transporters to their adaptability and invasiveness. Phylogenetic analysis categorized the 55 *B. tabaci* ABC transporters into eight subfamilies (Fig. 1). To further understand the evolutionary placement of *B. tabaci* ABC transporters, in-depth phylogenetic analyses were conducted for each subfamily. The results of these analyses were described and discussed in the following sections.

**Table 1:** ABC transporters identified in the *B. tabaci* Q genome

Subfamily	Gene ID	Length (aa)	Location (strand)	Exon	Topology (SMART)	Matched gene (Accession no.)	N-Glc^a^	O-Glc^b^	CDS status^c^
ABCA	Btabq017043.1	1655	Scaffold_3468:1006:32679+	20	TMD-NBD-TMD-NBD	ABCA3 (AIN44094.1)	10	7	C
	Btabq003223.3	1387	Scaffold_13:22456:46016-	18	TMD-NBD-TMD-NBD	ABCA3 (XP_008199154.1)	7	12	C
	Btabq003232.1	1710	Scaffold_13:231650:263916+	22	TMD-NBD-TMD-NBD	ABCA3 (XP_008580143.1)	13	13	C
	Btabq009375.1	1607	Scaffold_204:588616:628011+	24	TMD-NBD-TMD-NBD	ABCA3 (XP_004627914.1)	11	11	C
	Btabq009376.1	1642	Scaffold_204:635079:664477:-	24	TMD-NBD-TMD-NBD	ABCA3 (XP_007499572.1)	6	14	C
	Btabq022409.1	1642	Scaffold_499:371203:397123:+	23	TMD-NBD-TMD-NBD	ABCA3 (XP_007499572.2)	6	16	C
	Btabq022410.1	1653	Scaffold_499:405484:424414:-	21	TMD-NBD-TMD-NBD	ABCA3 (XP_007499572.3)	11	15	C
	Btabq008198.1	1730	Scaffold_188:144605:225978+	25	TMD-NBD-TMD-NBD	ABCA5 (XP_015513231.1)	11	25	C
ABCB	Btabq001304.1	837	Scaffold_11:1763748:1779600:-	13	TMD-NBD	ABCB6 (KDR08849.1)	5	0	C
	Btabq028971.1	684	Scaffold_89:122752:135916:+	13	TMD-NBD	ABCB7 (XP003707011.1)	3	12	C
	Btabq013065.1	903	Scaffold_266:334207:358911:-	19	TMD-NBD	ABCB8 (KDR20884.1)	2	8	C
ABCC	Btabq000311.1	1543	Scaffold_102:620714:659592:+	28	TMD-NBD-TMD-NBD	ABCC1 (XP_012252456.1)	8	10	C
	Btabq019529.2	1457	Scaffold_400:253922:298887:+	30	TMD-NBD-TMD-NBD	ABCC1 (KYM99989.1)	11	5	C
	Btabq026695.3	1422	Scaffold_728:117063:145027:+	27	TMD-NBD-TMD-NBD	ABCC4 (XP_003487856.1)	8	11	C
	Btabq008004.1	1302	Scaffold_1847:75114:101542:-	21	TMD-NBD-TMD-NBD	ABCC4 (XP_015366071.1)	11	4	C
	Btabq004618.1	1527	Scaffold_1440:62283:85796:-	24	TMD-NBD-TMD-NBD	ABCC7 (KDR17053.1)	11	3	C
	Btabq003933.1	1558	Scaffold_137:590708:610342:+	25	TMD-NBD-TMD-NBD	ABCC9 (XP_012225332.1)	9	3	C
ABCD	Btabq026746.1	733	Scaffold_732:28889:99259:+	4	TMD-NBD	ABCD2 (AIN44106.1)	3	5	C
	Btabq017051.1	670	Scaffold_347:310155:194947:-	15	TMD-NBD	ABCD3 (XP_008186233.1)	4	9	C
ABCE	BtabqABCE1	608	Scaffold_2889:666:11372:+	10	NBD-NBD	ABCE1 (XP_014275278.1)	1	5	C
ABCF	Btabq009873.1	595	Scaffold_2118:27608:29395:+	1	NBD-NBD	ABCF1 (XP_971562.1)	5	0	C
	Btabq014578.1	626	Scaffold_354:220262:229341:+	10	NBD-NBD	ABCF2 (XP_966990.1)	4	2	C
	Btabq016264.1	746	Scaffold_3286:21844:40980:-	13	NBD-NBD	ABCF3 (KDR22359.1)	3	5	C
ABCG	Btabq003568.1	679	Scaffold_133:520218:533101:-	14	NBD-TMD	ABCG1 (KDR13078.1)	6	0	C
	Btabq006006.1	634	Scaffold_1598:84277:126599:+	11	NBD-TMD	ABCG1 (XP_001943523.1)	1	4	C
	Btabq007377.1	594	Scaffold_176:564047:611847:-	11	NBD-TMD	ABCG1 (XP_001947776.2)	1	10	C
	Btabq020594.1	703	Scaffold_434:86892:119931:-	14	NBD-TMD	ABCG1 (XP_001942514.1)	1	6	C
	Btabq029952.1	654	Scaffold_973:236:11383:+	9	NBD-TMD	ABCG1 (KDR12258.1)	1	6	C
	Btabq009742.1	300	Scaffold_434:92701:149213:-	6	NBD	ABCG1 (XP_008476671.1)	1	3	P
	Btabq029281.1	676	Scaffold_910:266071:279898:-	12	NBD-TMD	ABCG4 (XP_003246360.1)	6	0	C
	Btabq007131.3	649	Scaffold_1729:110948:148435:+	13	NBD-TMD	ABCG4 (XP_008472465.1)	2	4	C
	Btabq023008.1	590	Scaffold_523:150957:167280:-	11	NBD-TMD	ABCG4 (XP_003246361.1)	2	6	C
	Btabq009608.1	590	Scaffold_208:388361:402826:-	10	NBD-TMD	ABCG4 (XP_003246361.1)	2	6	C
	Btabq009611.1	590	Scaffold_208:557522:571601:+	11	NBD-TMD	ABCG4 (XP_003246361.1)	2	6	C
	Btabq023919.1	639	Scaffold_563:13213:51650:-	10	NBD-TMD	ABCG4 (XP_008470161.1)	3	9	C
	Btabq013894.1	590	Scaffold_282:256960:283064:+	11	NBD-TMD	ABCG4 (XP_003246361.1)	2	6	C
	Btabq000844.1	655	Scaffold_1057:77963:94241:+	10	NBD-TMD	ABCG4 (XP_015509620.1)	2	0	C
	Btabq026080.1	692	Scaffold_683:129726:137969:+	3	NBD-TMD	ABCG4 (KDR08336.1)	6	11	C
	Btabq020567.1	692	Scaffold_4326:21216:33527:+	4	NBD-TMD	ABCG4 (KDR08336.1)	6	11	C
	Btabq001288.1	612	Scaffold_11:1463363:1478800:-	10	NBD-TMD	ABCG5 (KDR20187.1)	2	19	C
	Btabq001290.1	952	Scaffold_11:1493109:1530869:+	15	TMD	ABCG8 (XP_001944045.2)	4	19	P
	Btabq023890.1	755	Scaffold_561:73328:85538:-	15	NBD-TMD	ABCG14 (XP_014258500.1)	3	1	C
	Btabq014028.1	607	Scaffold_2842:67736:80804:+	12	NBD-TMD	Scarlet (XP_014276471.1)	3	11	C
	Btabq015484.1	645	Scaffold_3117:65588:78996:+	14	NBD-TMD	Scarlet (XP_014279356.1)	5	9	C
	Btabq002474.1	667	Scaffold_121:146665:158695:-	11	NBD-TMD	Scarlet (NP_001306193.1)	3	6	C
	Btabq022510.1	700	Scaffold_50:595327:617912:+	13	NBD-TMD	White (KDR20987.1)	3	8	C
ABCH	Btabq015123.1	685	Scaffold_304:505925:535800:-	15	NBD-TMD	ABCG23 (XP_001945365.2)	6	2	C
	Btabq003158.1	627	Scaffold_129:187020:214919:-	14	NBD-TMD	ABCG23 (XP_001945365.2)	4	1	C
	Btabq006712.2	687	Scaffold_167:585953:624494:-	16	NBD-TMD	ABCG23 (XP_001945365.2)	6	2	C
	Btabq018898.1	679	Scaffold_386:127646:145952:-	15	NBD-TMD	ABCG23 (XP_001945365.2)	4	6	C
	Btabq019352.1	685	Scaffold_398:364203:394966:+	15	NBD-TMD	ABCG23 (XP_015364832.1)	6	2	C
	Btabq026264.1	647	Scaffold_7:597935:614293:-	14	NBD-TMD	ABCG23 (XP_001942931.1)	5	1	C
	Btabq028063.1	749	Scaffold_82:494722:529828:-	16	NBD-TMD	ABCG23 (XP_001945213.1)	6	11	C
	Btabq009745.1	695	Scaffold_21:1506328:1537013:-	12	NBD-TMD	ABCG20 (XP_014289229.1)	5	3	C
	BTA027409.1	764	Scaffold_791:944:21302:+	16	NBD-TMD	ABCG20 (XP_001951744.1)	6	8	C

^a^N-glycosylation sites were predicted using the NetNGlyc 1.0 server (http://www.cbs.dtu.dk/services/NetNGlyc/); only N-glycosylation sites with a “potential” score > 0.5 and with a jury agreement were considered

^b^O-glycosylation sites were predicted using the NetOGlyc 4.0 server (http://www.cbs.dtu.dk/services/NetOGlyc/) [96]; if the G-score was > 0.5, the residue was considered to be O-glycosylated; this table shows the total number of O-glycosylated sites (glycosylated serines and threonines)

^c^C indicates a complete sequence; P indicates a partial sequence

**Table 2:** The distribution of ABC transporter subfamilies in *B. tabaci* Q and in other arthropod species

	Phloem-sucking			Blood-sucking			Chewing/Sucking				Chewing
Subfamily	*B. tabaci*	*D. noxia*	*T. urticae*	*An. gambiae*	*P. humanus*	*C. lectularius*	*A. mellifera*	*D. melanogaster*	*B. mori*	*H. armicoverpa*	*T. castaneum*
ABCA	8	3	9	9	2	6	3	10	6	7	10
ABCB	3	6	4	5	6	7	7	8	8	11	6
ABCC	6	24	39	13	5	6	9	14	15	11	35
ABCD	2	3	2	2	2	2	2	2	2	2	2
ABCE	1	1	1	1	1	1	1	1	1	1	1
ABCF	3	3	3	3	3	4	3	3	3	3	3
ABCG	23 (42%)	26 (32%)	23 (23%)	16 (31%)	13 (33%)	23 (45%)	15 (35%)	15 (27%)	13 (26%)	16 (30%)	13 (18%)
ABCH	9 (16%)	11 (13%)	22 (21%)	3 (6%)	6 (15%)	2 (3.9%)	3 (7%)	3 (5.3%)	3 (5.8%)	3 (5.5%)	3 (4%)
Others		5			2						
Total	55 (100%)	82 (100%)	103 (100%)	52 (100%)	40 (100%)	51 (100%)	43 (100%)	56 (100%)	51 (100%)	54 (100%)	73 (100%)

Species in this survey included *Bemisia tabaci, Diuraphis noxia, Tetranychus urticae, Anopheles gambiae, Pediculus humanus humanus, Cimex lectularius, Bombyx mori, Helicoverpa armigera, Apis mellifera, Drosophila melanogaster,* and *Tribolium castaneum.* “Others” refers to uncharacterized genes. The values without parentheses indicate the number of transporters detected; for subfamilies ABCG and ABCH, the values in parentheses indicate the percentage of all ABC transporters represented by the indicated subfamily

#### ABCA subfamily

A phylogeny tree of the eight ABCA proteins identified in *B. tabaci* and other organisms was presented in Additional file 2: Figure S2. All of the *B. tabaci* ABCA proteins are full-transporters (Table 1), which is consistent with *D. pulex* and *T. urticae* [10, 13]. The silkworm ABCA subfamily, in contrast, contains two full-transporters, one half-transporter and three incomplete ABC transporters [8]. Furthermore, no ABCA protein has been identified in yeast [26].

The *B. tabaci* ABCA subfamily has one of the largest *B. tabaci* ABC transporters, Btabq008198.1, encoding 1730 amino acids (Table 1). Btabq008198.1 clustered with the ABCA5 group, including *Anopheles gambiae* AGAp010416-PA, *D. noxia* DnXP_015375463.1 and *H. sapiens* ABCA5 cluster (hABCA5, 6, 8, 9 and 10) (Additional file 2: Figure S2). Although these ABCA5-related genes in mammalians (human, dog and mouse) cluster with ABCA5 on the same chromosomes [6, 27], no counterpart of the ABCA5-related genes has been identified in invertebrates, such as *B. mori* [8] and *T. urticae* [10]. Besides Btabq008198.1, the other seven *B. tabaci* ABCA genes (Btabq017043.1, Btabq003223.3, Btabq003232.1, Btabq009375.1, Btabq009376.1, Btabq022409.1, and Btabq022410.1) form a sister-group with a high bootstrap support. This sister-group clustered with *D. noxia* DnXP_015366480.1 (ABCA3), *T. japonicas* TjABCA3 and seven *T. urticae* genes. These genes then clustered with *D. melanogaster* DmCG31731 with a moderate bootstrap support. DmCG31731 was down-regulated in the salivary glands of a *D. melanogaster* E93 mutant [28]. As an early ecdysone responsive gene, E93 was a primary regulator of programmed cell death in *D. melanogaster* development [29]. Injection of dsRNAs synthesized from the two ABCA genes, TcABCA-9A and TcABC A-9B, led to approximately 30% mortality in the red flour beetle, *T. castaneum* [17].

#### ABCB subfamily

In *B. tabaci* Q, we identified three ABCB half-transporters but no full-transporters (Table 1). These *B. tabaci* half-transporters (Btabq001304.1, Btabq013065.1 and Btabq013065.1) belong to three clades, indicating that these genes are homologous to ABCB genes in human, fruit fly and other insects (Additional file 3: Figure S3). Btabq001304.1 clustered with ABCB6 proteins from other species, including *D. noxia* DnXP_015376167.1, *An. gambiae* AGAP002278*, D. melanogaster* DmCG4225, *T. japonicas* TjABCB6*,* and *H. sapiens hABCB6*. Similarly, Btabq028971.1 formed an ABCB7 clade with *D. noxia* DnXP_015378905.1, *D. melanogaster* DmCG7955, *T. japonicas* TjABCB7, and *H. sapiens hABCB7*. Meanwhile, Btabq013065.1 was clustered with ABCB8 from *D. melanogaster* (DmCG1824), *T. urticae* (Tu17g02000), *T. japonicas* (TjABCB8) and *H. sapiens* (hABCB8)*.*

In *H. sapiens*, hABCB6, hABCB7 and hABCB8/MABC1 are mitochondrial transporters with roles in iron metabolism and transportation of Fe/S protein precursors [30, 31]. *Drosophila melanogaster* DmCG4225 (ABCB6) was tolerant to cadmium [32]. Disruption of *C. elegans* ABTM- 1, a homologue of ABCB7, induced oxidative stress and premature cell death [33]. Furthermore, a homologous gene of *H. sapiens* ABCB7 in *Aedes aegypti* was up-regulated in an insecticide resistant strain [34]. Disruption of mouse ABCB8 decreased mitochondrial iron export and led to cardiomyopathy [35]. Similar to *D. pulex*, *B. tabaci* Q do not have homologous genes of human ABCB half-transporters that were associated with antigen processing (hABCB2, hABCB3, and hABCB9) [13].

#### ABCC subfamily

Insects typically have different number of ABCC transporters (Table 2). In *B. tabaci* Q, we identified six ABCC full-transporters, including Btabq019529.2, Btabq000311.1, Btabq008004.1, Btabq026695.3, Btabq004618.1 and Btabq003933.1. ABCC subfamily proteins are full-transporters with diverse functions, such as cell-surface receptor, ion transport and translocation of a broad range of substrates including drugs and endogenous compounds [36–39]. Due to their diverse functions, human ABCC subfamily members refer to multidrug resistance associated proteins (MRPs), which function as transporters; the cystic fibrosis transmembrane conductance regulator (CFTR, ABCC7), which forms a chloride channel; and sulfonylurea receptors (SUR1/2, ABCC8/9), which function as regulators of potassium channels [40]. In *H. sapiens*, “long” MRPs like ABCC1, 2, 3, 6 and 10 (MRP1, 2, 3, 6 and 7, respectively) have a TMD0, the third N-terminal transmembrane-spanning domain, while “short” MRPs like ABCC4, 5, 11 and 12 (MRP4, 5, 8 and 9, respectively) lack TMD0 [40].

In our phylogenetic analysis, two *B. tabaci* ABCC1 genes, Btabq019529.2 and Btabq000311.1, clustered with *D. noxia* DnXP_015366208.1, DnXP_015366209.1, *D. melanogaster* DmCG6214, *T. japonicus* ABCC1 and a group of *H. sapiens* “long” MRPs (MRP1, 2, 3 and 6) (Additional file 4: Figure S4). MRP1/ABCC1 transports a structurally diverse range of endogenous substances (e.g. leukotrienes and estrogen conjugates), xenobiotics and their metabolites [41]. These MRPs have been extensively studied as transporters of natural product drugs such as anthracyclines and plant alkaloids [42]. As a homologous gene to *H. sapiens* MRP1, MRP2, MRP3 and MRP6, DmCG6214 functions as a high capacity ATP-dependent organic anion transporter and acts as a transporter of ecdysteroid and juvenile hormone conjugates [43]. The other two ABCC genes in *B. tabaci,* Btabq008004.1 and Btabq026695.3, gathered with a large clade of ABCC4 in *D. noxia*, *An. gambiae* and a cluster of *Drosophila* ABCC proteins, including DmCG10505, DmCG14709 and others. MRP4/ABCC4 can remove a wide range of endogenous and exogenous molecules from cells [44]. The ABCC transporter encoded by CG10505 in *D. melanogaster* was regulated by heavy metals and was involved in biochemical detoxification of zinc and copper [45]. *Drosophila* DmCG14709 gene, a homologue of *H. sapiens* MRP4/ABCC4, was tightly regulated by oxygen [46]. The human body louse ABCC4 gene, *PhABCC4*, was up-regulated after exposed to the pesticide ivermectin. Injection of female lice with *PhABCC4* dsRNA significantly increased their sensitivity to ivermectin [47].

Btabq004618.1 clustered with *D. noxia* DnXP_015373973.1, *T. japonicas* TjABCC7, *D. melanogaster* DmCG7806, *H. sapien* hABCC10/MRP7 and *T. urticae* Tu03g07840. hABCC10/MRP7 was able to transport amphipathic anions and conferred resistance to some antitumor drugs [48]. In addition, Btabq003933.1 clustered with sulfonylurea receptors (SURs) in *D. noxia*, *D. melanogaster*, *T. japonicas*, *T. urticae* and *H. sapiens*. Unlike other ABCC subfamily genes, SURs help form ATP-sensitive potassium (K_AT P_) channels [38, 49]. Although SUR was considered to be a direct target of chitin synthesis inhibitors [50], it did not express in *D. melanogaster* epidermis, where chitin disruption was observed [51]. Moreover, data obtained from *D. melanogaster* suggested that chitin synthesis was independent of SUR [52]. Similarly, RNAi knockdown of SUR homologue in *T. castaneum* did not result in a phenotype [17]. ABCC subfamily is not only involved in detoxification and multidrug resistance in *Manduca sexta* but also participate in host plant selection in *T. urticae* [10, 53].

#### ABCD subfamily

*Bemisia tabaci* Q has two ABCD transporters, Btabq026746.1 and Btabq017051.1 (Table 1). The same amount of ABCDs have also been found in other insects [8], while more ABCDs were identified in *D. pulex, H. sapiens* and *C. elegans*. Members of ABCD subfamily are half-transporters and involve in the import of fatty acids and acyl-CoAs into this organelle [55]. ABCD transporters participated in peroxisome-related developmental progress in *C. elegans* [56]. Mutations in hABCD1/ALDP gene resulted in adrenoleukodystrophy, a genetic disorder that occurs primarily in males [57]. Our phylogenetic analysis suggested that three clades of ABCDs in metazoans, with the two *B. tabaci* ABCDs distributed in two clades (Additional file 5: Figure S5). Btabq026746.1 clustered with *D. noxia,* DnXP_015374078.1, *An. gambiae* AGAP002071, *D. melanogaster* DmCG216, *T. japonicas TjABCD2, H. sapiens hABCD2* and *hABCD1*. Meanwhile, Btabq017051.1 belongs to ABCD3 clade.

#### ABCE and ABCF subfamilies

ABCE and ABCF subfamilies contain atypical ABC transporters characterized by a pair of linked NBDs with no TMDs [1]. Their structure implied their roles in biological processes other than transportation. ABCE1 is one of the most evolutionarily conserved proteins and expressed in all organisms except eubacteria. Because of its role in translation and ribosome biosynthesis, ABCE1 is essential for all life stages [58]. *Homo sapiens* ABCE1/RNase L was initially identified as an inhibitor of RNase L [58]. Members of the ABCE subfamily in human and yeast also have a role in translation initiation [59]. However, the ABCE and ABCF transporters have not been well characterized in invertebrates. RNAi knockdown of an ABCE gene, TcABCE-3A, resulted in significant mortality in penultimate *T. castaneum* larvae [17]. ABCE subfamily has only one member in all metazoans studied to date [9, 60], and *B. tabaci* follows this rule (BtabqABCE1, Table 1 and Additional file 6: Figure S6).

For ABCF subfamily, three transporters, Btabq009873.1, Btabq014578.1, and Btabq016264.1, were annotated and showed well-supported sister clades (Additional file 7: Figure S7). Btabq009873.1, Btabq014578.1, and Btabq016264.1 were placed within the ABCF1, ABCF2, and ABCF3 clades, respectively. In human and yeast, ABCF proteins participated in gene regulation systems and ribosome assembly [14]. Mutations in the yeast GCN20 gene, which was involved in the initiation and control of translation, reduced Eif2α phosphorylation and translation in the ribosome [61]. Knockdown of TcABCF-2A led to 100% mortality in penultimate *T. castaneum* larvae [17].

#### ABCG subfamily

ABCGs have a typical reverse domain architecture, with a NBD localized at the N-terminus and a TMD at the C-terminus (NBD-TMD). The ABCG transporters in metazoans are half-transporters and can be a functional transporter only after dimerization. In plants and fungi, however, ABCGs are full-transporters, also called pleiotropic drug resistance proteins (PDRs) [16, 62]. Because of their duplicated domain structure, yeast PDRs were not included in the phylogenetic analyses in this study.

ABCG proteins represent the largest ABC subfamily (23 members) in *B. tabaci* (Table 1) and also form the largest ABC subfamily in *D. noxi*a [63], *An. gambiae* [7]*,* bed bug (*Cimex lectularius*) [64], human body louse (*Pediculus humanus humanus*) [65], *Drosophila* [1] and *A. mellifera* [8] (Table 2). According to Sturm *et al.* [13], the expansion of ABCG transporters in *D. pulex* and *D. melanogaster* genomes was resulted from extensive lineage specific gene duplications.

Among the 23 ABCG members in *B. tabaci* Q, 17 are ABCG1 and ABCG4 transporters*.* These ABCG1 and ABCG4 proteins clustered with those in *D. noxia*, *An. gambiae*, *T. urticae,* and *D. melanogaster* (DmCG9664, DmCG32091, DmCG4822, DmCG31689, DmCG9663, DmCG17646, DmCG5853 and DmCG3164) (Additional file 8: Figure S8). The ABCG4 gene has the same intron/exon structure as ABCG1, suggesting that they arose by a relatively recent gene duplication event [66]. Similar to ABCG1, ABCG4 can be inducible by oxysterols and retinoids [67]. In *H. sapiens*, hABCG1 and hABCG4 were involved in cellular cholesterol efflux [68]. In addition, the elevated expression of ABCG4 in *An. stephensi* suggested its involvement in the degradation of a pyrethroid insecticide [18, 69].

Btabq001288.1 and Btabq001290.1 clustered with DnXP_01579755.1, DnXP_015379710.1, DmCG11069, DmCG31121, Tu01g16280, TjABCG5, hABCG5 and hABCG8. hABCG5 and hABCG8 mediated the intestinal and biliary efflux of cholesterol and sterols [68]. A clear subclass was formed with Btabq023890.1, *D. melanogaster* DmCG3327, and *T. urticae* tetur17g02510. DmCG3327 (also named E23), a 20-hydroxyecdysone (20E)-induced ABC transporter, regulated metamorphosis, probably by removing 20E from cells [70]. Similarly, ABCG expression in silkworm midgut was also regulated by 20E [8]. RNAi-based functional study confirmed a similar role in metamorphosis of TcABCG-8A, a homologue of E23 in *T. castaneum* [17].

Among invertebrates, the functions of ABCGs were first characterized in *Drosophila* as pigment precursor transporters (brown, scarlet and white genes) [71, 72]. In our phylogenetic analysis, four *B. tabaci* ABCG proteins, Btabq014028.1, Btabq015484.1, Btabq002474.1 and Btabq022510.1, showed homologous relationships with these pigment precursor transporters in *D. noxia, An. gambiae* and *D. melanogaster*. Besides transporting pigment precursors, these transporters also function in courtship behavior [73], transport of biogenic amines [74] and up-take of uric acid [75]. RNAi-mediated suppression and genetic linkage analysis confirmed that down-regulation of *Pxwhite* gene was tightly linked to Cry1Ac resistance in *Plutella xylostella* [76].

Based on a recent review, 80% of the peer-reviewed publications involving arthropod ABC transporters (50) linked ABC transporters with insecticides-resistance [77]. Members of ABCG subfamily were over-expressed in thiamethoxam-resistant *B. tabaci*, suggesting the involvement of these genes in insecticide resistance [78]. In addition, ABCG subfamily not only participated in multi-pesticide resistance in *T. urticae* but also reduced the chemical defense from host plants [10]. In *B. mori,* at least five ABCG genes acted as 20-hydroxyecdysone (20E)-response genes and participated in hormonal regulation [8]. These results suggested that members of the ABCG subfamily play significant roles in the development and the removal of amphiphilic xenobiotics. Although ABC transporters in subfamilies B, C, and G are involved in the metabolic resistance to various xenobiotics, *B. tabaci* Q, however, contains predominantly G, but few B and C family members (Table 2). This bias reflects the importance of ABCG subfamily in *B. tabaci* to fend off xenobiotic offences derived from host plants as well as agroecosystems.

#### ABCH subfamily

As inverse half-transporters, ABCH proteins share the same domain architecture as ABCG transporters. ABCH transporters were first discovered in *D. melanogaster* and have only annotated in arthropods and zebrafish [6, 8, 10, 11, 13, 17]. ABCH proteins have not been identified in fungi, *C. elegans*, plants and mammals [1, 16, 54, 62]. ABCH subfamily was documented in zebrafish but not in catfish, fugu or cod [79].

A total of nine ABCH transporters were identified in *B. tabaci*. Phylogenetic analysis showed that ABCH subfamily members were species-specific. *Bemisia tabaci* ABCH clustered with genes in *D. noxia* with a high bootstrap value (Additional file 9: Figure S9). An RNAi screen of *D. melanogaster* genes revealed that silencing of *D. melanogaster* CG9990 was lethal [80, 81]. In two insecticide-resistant *P. xylostella* strains, an ABCH (*Px014955*), was the most up-regulated ABC transporter [82]. Knockdown of *TcABCH-9C*, an ABCH in *T. castaneum*, resulted in 100% larval mortality and a significant reduction of fecundity and hatching rate. In addition, *T. castaneum* larvae injected with *TcABCH-9C* dsRNA showed a lack of lipids in their epicuticle, indicating that *TcABCH-9C* may be required for the formation of a waterproof barrier in the epicuticle [17]. In the cotton bollworm *Helicoverpa armigera* and *Manduca sexta*, the expression of ABCH subfamily was highly induced after larvae fed with secondary metabolites [19, 53].

### Expression profiles across developmental stages

**Fig. 2:**
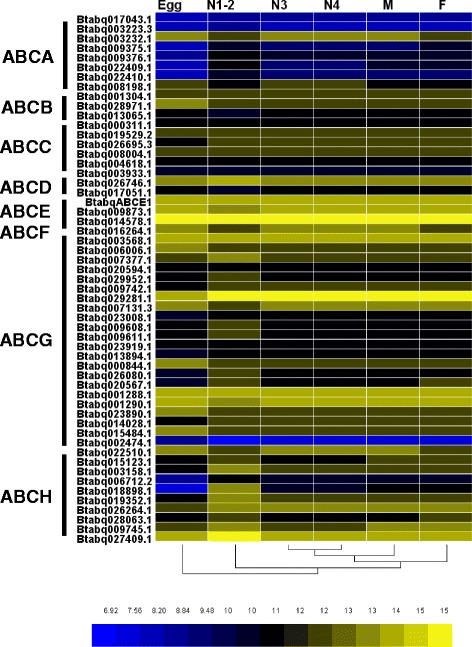
Expression profiles of ABC transporters throughout *B. tabaci* Q life cycle. The codes on the left are the gene ID numbers of the 55 ABC transporters in *B. tabaci* Q. The mRNA levels, as represented by log_2_ (RPKM + 1) values, are shown in the gradient heat map with colors ranging from blue (low expression) to yellow (high expression). For each stage, values are normalized data from three biological replicates. E, egg; N1-2, 1^st^- and 2^nd^-instar nymphs; N3, 3^rd^-instar nymph; N4, 4^th^-instar nymph; F, adult female; M, adult male

Based on RNA-seq data, all 55 ABC transporters were expressed with a RPKM (Reads Per Kilobases per Million reads) >1 in at least one of the *B. tabaci* life stages (Additional file 10: Table S1). Members from ABCE and ABCF subfamilies were ubiquitous and expressed highly throughout *B. tabaci* developmental stages (Fig. 2). In the polyphagous spider mite, *T. urticae*, an ABCE (*tetur30g01400*), showed high expression level throughout the life cycle [10]. Moreover, *TcABCE-3A* transcripts were abundant in all developmental stages of *T. castaneum* [17]. The expression profile of ABCE genes in the intertidal copepod, *T. japonicas,* and sea lamprey, *Petromyzon marinus,* showed similar trends as well [83, 84]. *Btabq014578.1* (ABCF2) had the highest expression level throughout the life cycle. ABCE1, ABCF1 and ABCF2 were highly expressed in all developmental stages of *B. tabaci*. ABCF3, also ubiquitously expressed, exhibited a lower expression level. These gene expression patterns were consistent with the fundamental functions in ribosome biogenesis and translation regulation [17, 85, 86]. The results suggest that ABCE and ABCF transporters were possibly essential to maintain “housekeeping” function in *B. tabaci*.

The expression levels for ABCA transporters in *B. tabaci* were relatively low with the exception of Btabq003232.1 (ABCA3), which was highly expressed in all life stages (Fig. 2). ABCA3 is critical for the proper formation of lamellar bodies and surfactant function and may participate in surfactant phospholipid metabolism [87]. Btabq001304.1 (ABCB6) expressed in all life stages of *B. tabaci* and highly expressed in eggs and nymphs. Btabq028971.1 (ABCB7) expressed in all developmental stages, especially in the fourth nymph stage and adult. Btabq013065.1 (ABCB8) expressed in all stages but at a lower level than other ABCB genes. *Homo sapiens* ABCB half-transporters, ABCB6, ABCB7, ABCB8 and ABCB10, were localized at mitochondria and functioned in iron metabolism [88].

Expression levels of all ABCC members were documented in each developmental stage of *B. tabaci* Q, and the expression patterns were different among these genes (Fig. 2). Only Btabq008004.1 was highly expressed throughout life stages (Fig. 2). The two *B. tabaci* ABCDs, Btabq026746.1 and Btabq017051.1, showed stable expressional level among different developmental stages (Fig. 2). Btabq026746.1 was highly expressed while Btabq017051.1 showed slight lower expression level. Because the two ABCDs are homologous to those of mammal and insect transporters, similar functions such as roles in fatty acid metabolism can be inferred in *B. tabaci* Q.

Members of the ABCG subfamily showed different expression patterns in *B. tabaci* Q*.* Btabq003568.1, Btabq029281.1 and Btabq007131.3 were ubiquitously with high expression profiles. Expression levels were also high for Btabq006006.1, Btabq007377.1, Btabq029952.1 and Btabq000844.1. Overall, ABCG1 and ABCG4 members in *B. tabaci* Q had moderate to high expressional levels in all developmental stages. The ABCG4 in *An. stephensi* showed an increased expressional level in defense against the pyrethroid insecticide [18, 69]. Btabq001288.1 (ABCG5) and Btabq001290.1 (ABCG8) showed high expression in all stages of *B. tabaci*. Homologues of ABCG5 and ABCG8 in *D. melanogaster* and *T. castaneum* function, respectively, in metamorphosis [17] and may have a similar function in *B. tabaci*. Furthermore, Btabq022510.1 was annotated as a *white* gene and its expression level in nymph and adult stages was especially high. Recently, a *Pxwhite* gene was shown to be tightly linked to Cry1Ac resistance in *P. xylostella* [76].

ABCH transporters differed in expression patterns during *B. tabaci* development. Btabq027409.1 and Btabq026264.1 were highly expressed in all developmental stages. Moreover, Btabq015123.1, Btabq009745.1, and Btabq019352.1 also showed high expression level, especially in the 4^th^-instar nymphs and adults. One ABCH subfamily gene, CG9990, was presumed to be involved in mortality [81]. Silencing of *T. castaneum TcABCH-9C* reduced oviposition and pupal molting [17]. ABCH transporters are highest expressed in the head of *H. armigera* when the larvae were ingested secondary metabolism [19].

Expansion of ABCG and ABCH subfamilies do exist in *B. tabaci* Q. Combined with their expressional profiles, we suspect that these ABCG and ABCH genes are involved in development-related metabolism and in the detoxification of xenobiotics including insecticides and secondary metabolites from host plant.

### mRNA expression of selected ABC transporters by RT-qPCR

**Fig. 3:**
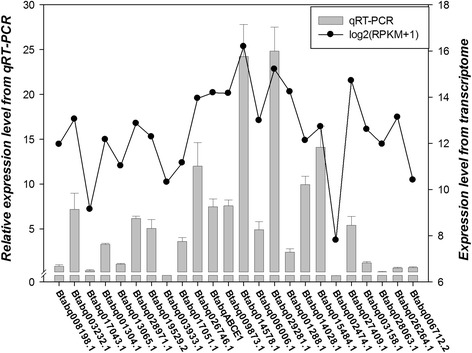
mRNA expressions of selected *B. tabaci* Q ABC transporters by RT-qPCR. The bars represent expression level of *B. tabaci* Q ABC transporters relative to the Btabq008198.1. Data are presented as means ± SE. Spots indicated log_2_ (RPKM + 1) values of ABC transporters in adults. Significant correlation between RT-qPCR and transcriptomic analyses were assessed by SPSS

The expression profiles of 24 selected ABC transporters were confirmed by the RT-qPCR (Fig. 3). Within ABCA subfamily, Btabq003232.1 had the highest expressional level, followed by Btabq008198.1 and Btabq017043.1. This trend is consistent with RNA-seq analysis. Similarly, gene expression level of ABCB genes from high to low was Btabq028971.2, Btabq001304.1 and Btabq013065.1. Expression profiles of ABCC, ABCD, ABCE and ABCF subfamilies were also consistent with our RNA-seq data. In ABCG subfamily, the trend persisted. Only two genes in ABCH subfamily, Btabq028063.1 and Btabaq026264.1, exhibited lower expressional level, which is not consistent with the RNA-seq data. Statistical analysis by SPSS demonstrated a significant correlation between transcriptome data and RT-qPCR data (*r* = 0.684, *p* < 0.01).

### Transcriptional response to imidacloprid treatments

**Fig. 4:**
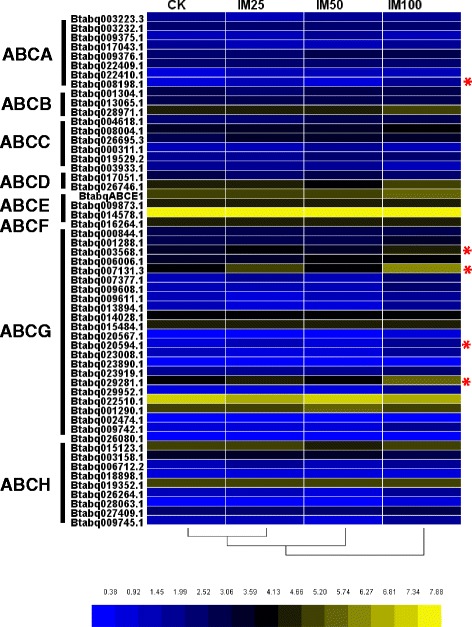
Transcriptional response of ABC transporters in *B. tabaci* Q adults to imidacloprid. The mRNA expression levels, i.e., log2 (RPKM + 1) values, are represented in a gradient heat map from low (blue) to high (yellow). Values were averaged from the three biological replications. The *B. tabaci* Q adults were fed with either water (CK) or imidacloprid at 25, 50, and 100 mg/L concentration (IM25, IM50, or IM100, respectively). An asterisk indicates a significant difference

A total of five genes were significantly up-regulated in imidacloprid-treated *B. tabaci* (Fig. 4, Additional file 11: Table S4). Btabq008198.1, an ABCA5 gene, can regulate accumulation of cholesterol [89]. The other four belong to ABCG subfamily, including Btabq003568.1 (ABCG1), Btabq020594.1 (ABCG1), Btabq007131.3 (ABCG4), and Btabq029281.1 (ABCG4).

The metabolic detoxification of xenobiotics likely involves a set of detoxification enzymes, which are general classified into three categories. Besides Phase I (e.g., cytochrome P450 monooxygenases) and Phase II (e.g., glutathione S-transferases), ABC transporters represent a major class of Phase III enzymes.

ABC transporters, especially those in the ABCG subfamily, facilitated *H. armigera* defense against secondary metabolites such as nicotine and tomatine [19]. The high expression of ABCG11 in *H. armigera* was the results of nicotine ingestion [19]. In addition, ABCG4 in *An. stephensi* was up-regulated in response to pyrethroid insecticide [18, 69]. The expression levels of three ABC transporters, ABCG20-3, ABCG23-5 and ABCH-B, were higher in field-collected resistant populations of *C. lectularius* [90]. Then knockdown of ABCG20-3 reduced deltamethrin resistance [90]. ABC transporters, particularly ABCG1 and ABCG4, may have important role in response to xenobiotics. Moreover, ABCG4 gene has the same intron/exon structure as ABCG1, suggesting that they arose by a relatively recent gene duplication event [66]. Overall, feeding on imidacloprid significantly affected the expression of ABC transporters especially those in ABCG subfamily, suggesting the potential involvement of these genes in the *B. tabaci* metabolic resistance.

## Conclusion

In this study, we identified 55 ABC transporters in the sweetpotato whitefly *B. tabaci* Q genome. ABC transporter subfamilies G and H are expanded in *B. tabaci* Q genome. The expression profiles of these ABC transporters were validated using both RT-qPCR and RNA-seq analyses. Furthermore, 80% of the significantly up-regulated ABC transporters in imidacloprid-challenged *B. tabaci* were ABCGs. These combined results imply the potential contribution of ABCGs to the adaptability of this emerging global invasive pest.

## Methods

### Colony maintenance and sample preparation

*Bemisia tabaci* Q biotype (Mediterranean, MED) population maintained on cotton plant at 27 ± 1 °C, with a photoperiod of 16 h light: 8 h darkness and 70 ± 10% relative humidity. Every 3–5 generations, the purity of the strain was monitored using a mitochondrial cytochrome oxidase I (mtCOI) marker [91].

The different developmental stage samples (eggs, four nymph stages, females and males) were collected from the Q biotype population described above. Newly emerged (within 5 days) adults of *B. tabaci* Q on cotton were collected to be treat with imidacloprid following adult leaf-dip bioassay method [92, 93]. In brief, leaf discs (22 mm in diameter) from cotton plants were dipped in imidacloprid at 0 (distilled water control), 25, 50 and 100 mg/L for 10s. When dry, the leaf discs were placed on agar in a flat-bottomed glass tube, 20 *B. tabaci* adults were added to each tube. After 48 h, adults were collected, snap frozen in liquid nitrogen, and transferred to −80 °C for the long-term storage. Three biological replications for all *B. tabaci* samples were carried out independently. A total of 100 individuals were collected for each biological replication, and these samples were subjected to RNA-seq analysis.

### Identification and annotation of ABC transporters

To identify open reading frames (ORFs) encoding putative ABC transporters, we carried out tblastn searches against the *B. tabaci* Q genome [94] and transcriptome assembled from SRP064690. We subsequently used the highly conserved NBDs (as defined by InterPro domain IPR003439/PF00005) and full-sequences of all 56 ABC genes in *D. melanogaster* as queries to search against the updated GLEAN gene in *B. tabaci* genome with an E-value threshold of 10^−5^ to identify ABC transporters. Each putative ABC transporter gene was confirmed by subjected it to BLASTX analysis with the non-redundant protein sequence (NR) at GenBank (http://www.ncbi.nlm.nih.gov/).

The exon/intron boundary and start/stop codons of each ABC gene were confirm from the genome and transcriptome. The conserved domains of these identified ABC proteins were predicted using SMART (simple modular architecture research tool, http://smart.embl-heidelberg.de/) [95] and confirmed by using the NCBI conserved domain search service tool. Putative N-glycosylation sites were predicted using NetNGlyc 1.0 server and O-glycosylation sites were predicted using NetOGlyc 4.0 server [96].

### Phylogenetic analyses

To analyze the evolutionary placement of ABC transporters in *B. tabaci*, we collected the readily available ABC transporters from the fruit fly, *D. melanogaster*, wheat aphid, *D. noxia*, mosquito, *An. gambiae*, spider mite, *T. urticae*, intertidal copepod, *T. japonicus*, human, *H. sapiens*, nematode, *Caenorhabditis elegans*, and yeast, *Saccharomyces cerevisiae*. NBDs of *B. tabaci* ABC transporters were used to resolve their phylogenetic relationships at the subfamily level. Within each ABC subfamily, we used the full-length protein sequences to construct their respective phylogeny trees [9, 10]. Additional file 12: Table S5 lists models of the constructed phylogenetic trees. Sequences of entire transporters aligned using ClustalW and then subjected to phylogenetic analysis by MEGA5 with 1000 bootstrap replications [97]. The phylogenetic trees were constructed using the maximum-likelihood method.

### Expression profiling of ABC transporters

Expression profiling of ABC transporters was assessed using transcriptome data of different developmental stages and imidacloprid-treated whitefly as described earlier. ABC genes were also verified by quantitative real-time reverse transcription polymerase chain reaction (RT-qPCR). Total RNA was extracted using Trizol reagent according to the manufacturer’s instructions (Invitrogen, Carlsbad, CA, USA). RNA was quantified using a Nanodrop 2000 (Thermo Scientific, Wilmington, DE, USA) meanwhile purity was checked on 1% agarose gels. RNA-seq libraries were constructed as previously described [98] and sequenced on a HiSeq 2500 system according to the manufacturer’s instructions with sequenced at 125 bp (PE125, library size is 280–320 bp). RNA-seq libraries of imidacloprid-treated samples were constructed by the same method and sequenced on a HiSeq 4000 system according to the manufacturer’s instructions with sequenced at 150 bp (PE150, library size is 450–550 bp). The raw reads were filtered with Fastq clean software [99] to trim low quality (Q value < 20) nucleotides on both ends, clipping the adapter and barcode sequences from the 3’ end and discarding the ribosomal RNA (rRNA) sequence. The high-quality cleaned reads were then aligned to the pre-prepared RNA sequence dataset with the Bowtie program [100] allowing one mismatch. After alignments, raw counts for each *B. tabaci* transcript and each sample were derived and were normalized to Reads Per Kilobase of transcript per Million mapped reads (RPKM). Differentially expressed genes (fold changes > 2 and adjusted *P*-value < 0.05) were identified by the DESeq package [101–103].

Besides transcriptomic validation, mRNA expressions of ABC transporters in *B. tabaci* Q biotype were confirmed using RT-qPCR analysis as well. Based on the RPKM value generated from the RNA-seq data, we selected 24 transcripts representing all eight subfamilies of the *B. tabaci* Q biotype ABC transporters for the RT-qPCR validation study (Additional file 13: Table S2 and Additional file 14: Table S3). RT-qPCR was conducted using an ABI PRISM 7500 Real-time PCR System (Applied Biosystems, Foster, CA, USA), and non-treated *B. tabaci* adults were subjected to the analysis. All RT-qPCR analyses included three technical replicates for each of three biological replicates. *Elongation factor 1* (EF1) and *TAF10 RNA polymerase II* (TAF) were selected as reference genes [104, 105]. The RT-qPCR was carried out in a 25 μL reaction volume containing 12.5 μL 2 × SuperReal PreMix Plus, 0.5 μL 50 × ROX Reference Dye, 0.75 μL forward primer (10 μM), 0.75 μL reverse primer (10 μM), 1.0 μL cDNA (300 ng/μL), and 9.5 μL RNase-free ddH2O, following the instructions of the SuperReal PreMix Plus (SYBR Green) kit (Tiangen, Beijing, China). The thermal cycling conditions were polymerase activation at 95 °C for 15 min, followed by 40 cycles of denaturation at 95 °C for 10 s, annealing at 60 °C for 30 s and elongation at 72 °C for 32 s. The amplification efficiency was estimated using the equation: E = [10^(−1/slope) − 1] × 100%, in which the slope was derived from plotting the cycle threshold (Ct) value versus six serially diluted template concentrations. The transcript levels of ABC genes were quantified according to the 2^−ΔΔCt^ method [106]. SPSS 19.0 was used to analyze correlations between RT-qPCR data and RNA-seq data.

## Additional files

Additional file 1: Figure S1. Comparison of gene numbers in each subfamily of ABC transporters between *Bemisia tabaci* and other organisms. (PDF 23 kb)

Additional file 2: Figure S2. Phylogenetic relationship of *Bemisia tabaci* ABCA subfamily with other organisms. Full-length ABC transporters were aligned using ClustalW and subjected to a maximum likelihood analysis by MEGA5 [97]. Numbers at the branch point of the node represent the values resulting from 1000 replications. Species, abbreviations, and color codes are: Btabq, *B. tabaci* (red); Dn, *D. noxia* (blue); AGA, *A. gambiae* (green); Dm, *D. melanogaster* (purple); Tu, *T. urticae* (light blue); Tj, *T. japonicus* (black); h, *H. sapiens* (gray); Ce, *C. elegans* (brick-red); Sc, *Saccharomyces cervisiae* (orange). (PDF 28 kb)

Additional file 3: Figure S3. Phylogenetic relationship of *Bemisia tabaci* ABCB subfamily with other organisms. See Figure S2 legend for details. (PDF 14 kb)

Additional file 4: Figure S4. Phylogenetic relationship of *Bemisia tabaci* ABCC subfamily with other organisms. See Figure S2 legend for details. (PDF 15 kb)

Additional file 5: Figure S5. Phylogenetic relationship of *Bemisia tabaci* ABCD subfamily with other organisms. See Figure S2 legend for details. (PDF 19 kb)

Additional file 6: Figure S6. Phylogenetic relationship of *Bemisia tabaci* ABCE subfamily with other organisms. See Figure S2 legend for details. (PDF 37 kb)

Additional file 7: Figure S7. Phylogenetic relationship of *Bemisia tabaci* ABCF subfamily with other organisms. See Figure S2 legend for details. (PDF 89 kb)

Additional file 8: Figure S8. Phylogenetic relationship of *Bemisia tabaci* ABCG subfamily with other organisms. See Figure S2 legend for details. (PDF 16 kb)

Additional file 9: Figure S9. Phylogenetic relationship of *Bemisia tabaci* ABCH subfamily with other organisms. See Figure S2 legend for details. (PDF 134 kb)

Additional file 10: Table S1. RPKM values of *B. tabaci* Q ABC transporters at different developmental stages (DOCX 13 kb)

Additional file 11: Table S4. Fold-change of gene expression following *B. tabaci* Q exposure to imidacloprid (DOCX 8 kb)

Additional file 12: Table S5. Models used for phylogenetic analysis (DOCX 15 kb)

Additional file 13: Table S2. Selected *B. tabaci* Q ABC transporters for the RT-qPCR validation study (DOCX 143 kb)

Additional file 14: Table S3. Primers used for the RT-qPCR analysis (DOCX 89 kb)

## Abbreviations

20E: 20-hydroxyecdysone
ABC: ATP-binding cassette
CFTR: The cystic fibrosis transmembrane conductance regulator
MRP: Multidrug resistance associated protein
NBD: Nucleotide binding domain
PDR: Pleiotropic drug resistance protein
RPKM: Reads Per Kilobases of per Million reads
RT-qPCR: Quantitative real-time reverse transcription polymerase chain reaction
SUR: Sulfonylurea receptor
TMD: Transmembrane domain.
