# Supplementary material for: Genome-wide analysis of ATP-binding cassette (ABC) transporters in the sweetpotato whitefly, Bemisia tabaci
Source: BMC Genomics. 2017 Apr 26;18:330. doi: 10.1186/s12864-017-3706-6 (PMC5405539; doi:10.1186/s12864-017-3706-6)
Supplement: Supplementary file 1 — Comparison of gene numbers in each subfamily of ABC transporters between Bemisia tabaci and other organisms. (PDF 23 kb) [file 12864_2017_3706_MOESM1_ESM.pdf]

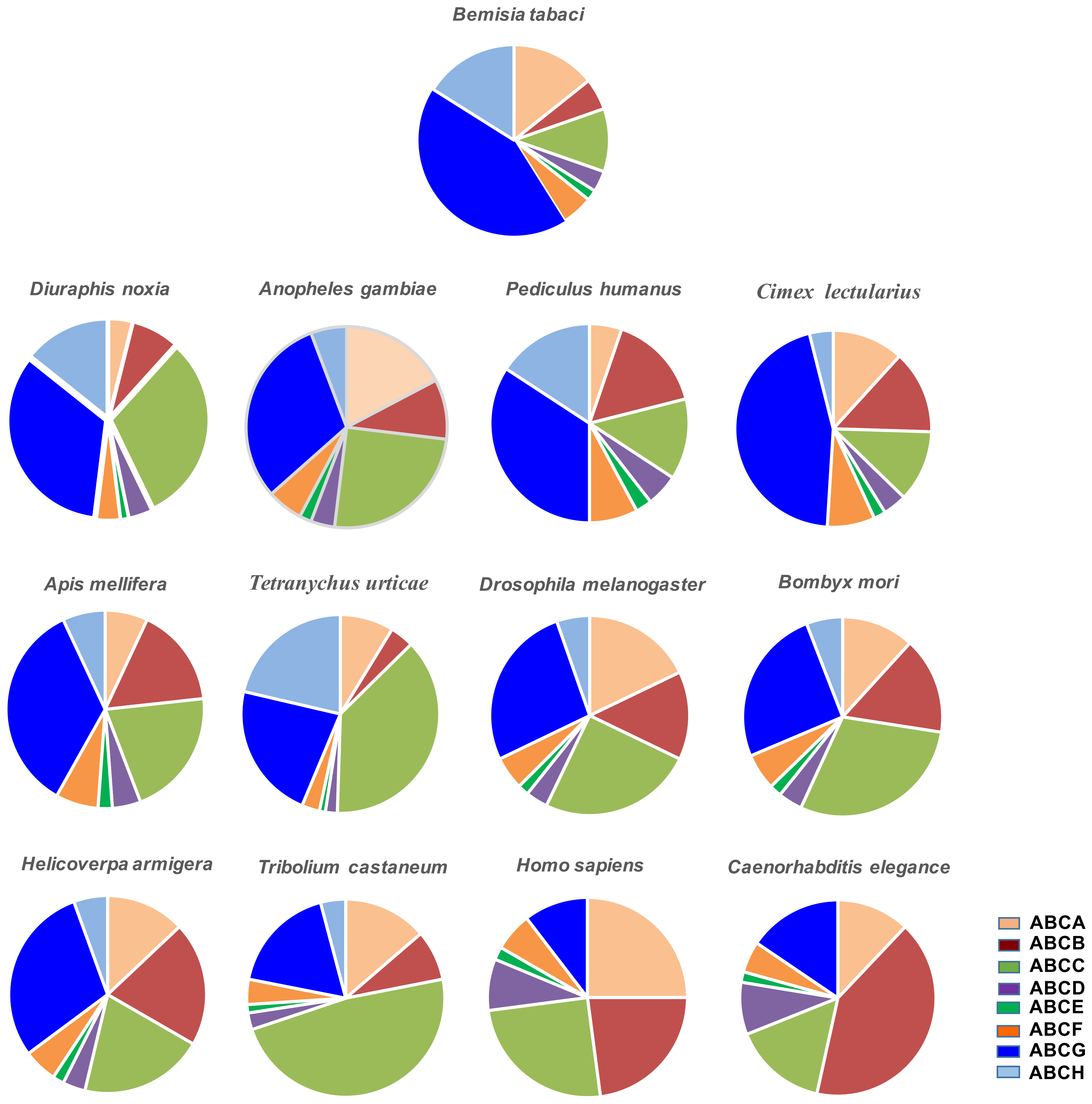

Bemisia tabaci
Diuraphis noxia
Anopheles gambiae
Pediculus humanus
Cimex lectularius
Apis mellifera
Tetranychus urticae
Drosophila melanogaster
Bombyx mori
Helicoverpa armigera
Tribolium castaneum
Homo sapiens
Caenorhabditis elegance
ABCA
ABCB
ABCC
ABCD
ABCE
ABCF
ABCG
ABCH
