# Supplementary figures and images for: Genome-wide analysis of ATP-binding cassette (ABC) transporters in the sweetpotato whitefly, Bemisia tabaci
Source: BMC Genomics. 2017 Apr 26;18:330. doi: 10.1186/s12864-017-3706-6 (PMC5405539; doi:10.1186/s12864-017-3706-6)

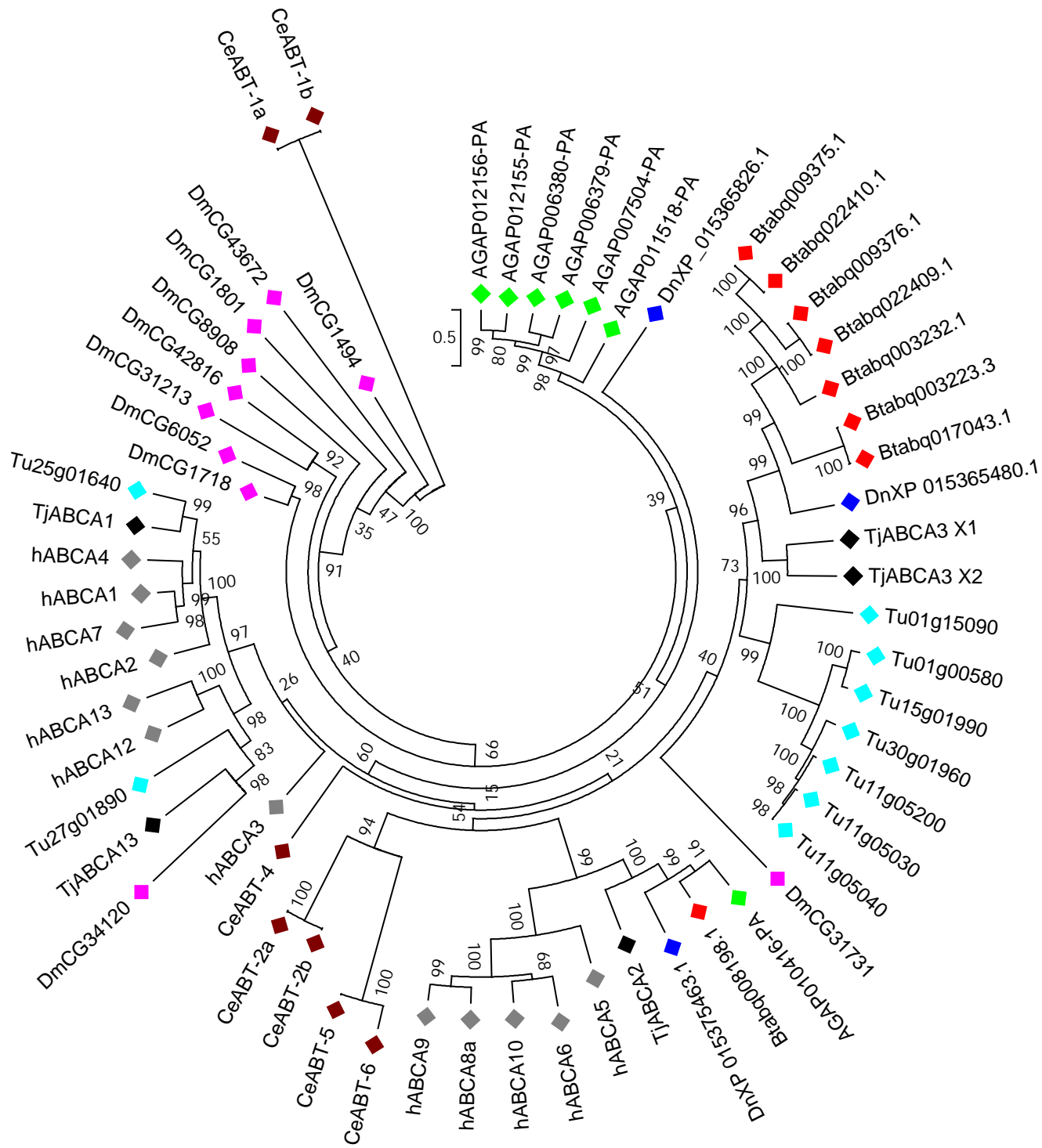

Supplement: Supplementary file 2 — Phylogenetic relationship of Bemisia tabaci ABCA subfamily with other organisms. Full-length ABC transporters were aligned using ClustalW and subjected to a maximum likelihood analysis by MEGA5 [97]. Numbers at the branch point of the node represent the values resulting from 1000 replications. Species, abbreviations, and color codes are: Btabq, B. tabaci (red); Dn, D. noxia (blue); AGA, A. gambiae (green); Dm, D. melanogaster (purple); Tu, T. urticae (light blue); Tj, T. japonicus (black); h, H. sapiens (gray); Ce, C. elegans (brick-red); Sc, Saccharomyces cervisiae (orange). (PDF 28 kb) [file 12864_2017_3706_MOESM2_ESM.pdf]

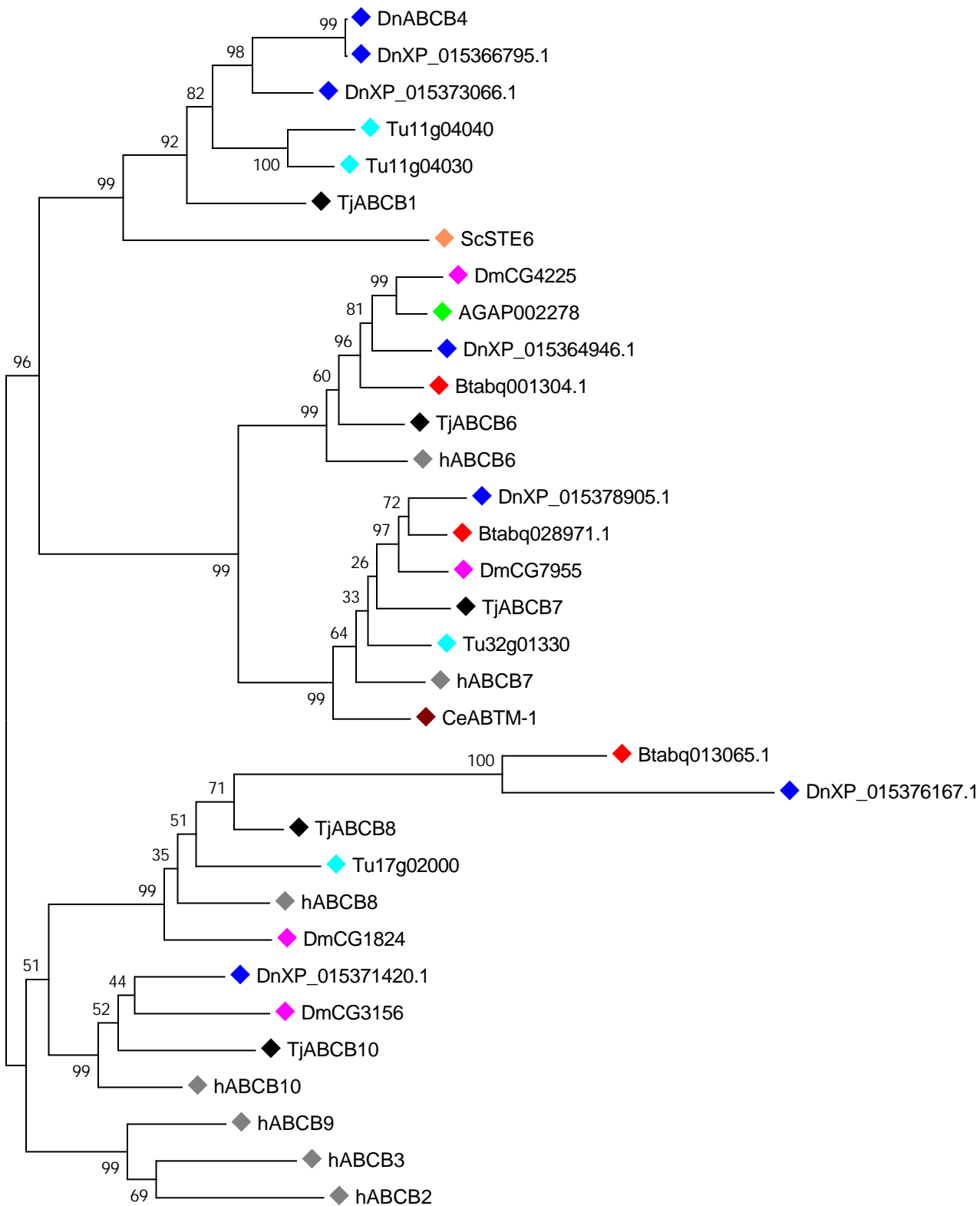

0.2

Supplement: Supplementary file 3 — Phylogenetic relationship of Bemisia tabaci ABCB subfamily with other organisms. See Figure S2 legend for details. (PDF 14 kb) [file 12864_2017_3706_MOESM3_ESM.pdf]

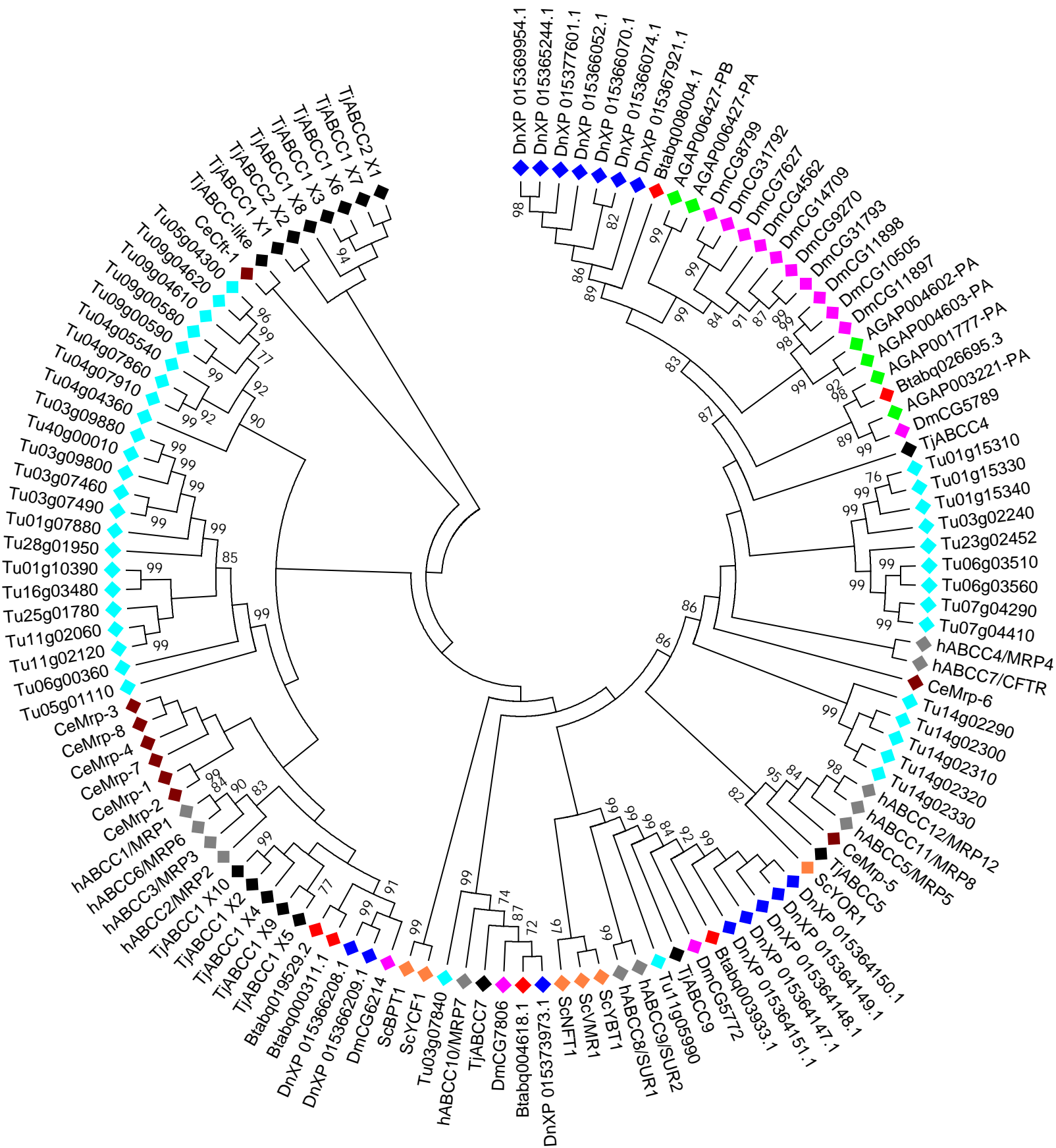

Supplement: Supplementary file 4 — Phylogenetic relationship of Bemisia tabaci ABCC subfamily with other organisms. See Figure S2 legend for details. (PDF 15 kb) [file 12864_2017_3706_MOESM4_ESM.pdf]

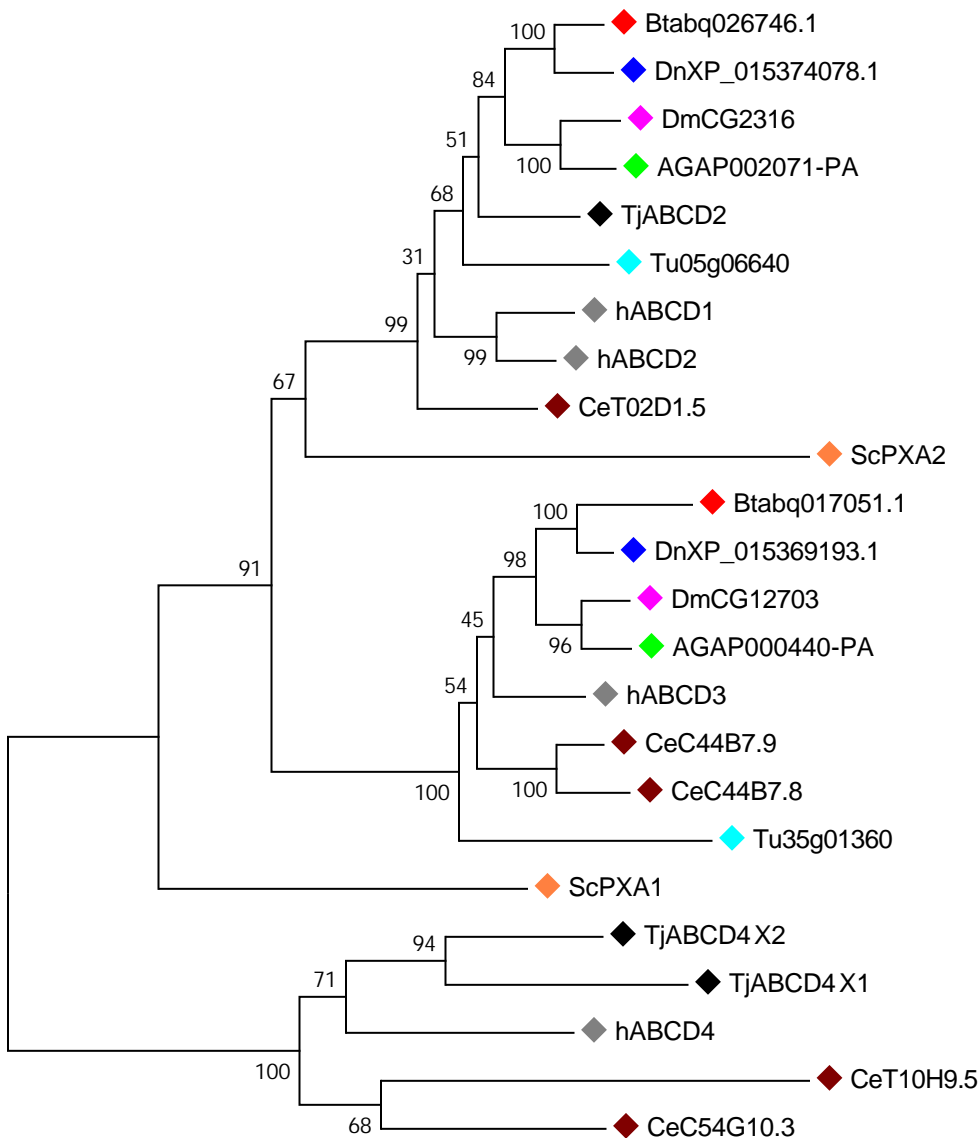

0.5

Supplement: Supplementary file 5 — Phylogenetic relationship of Bemisia tabaci ABCD subfamily with other organisms. See Figure S2 legend for details. (PDF 19 kb) [file 12864_2017_3706_MOESM5_ESM.pdf]

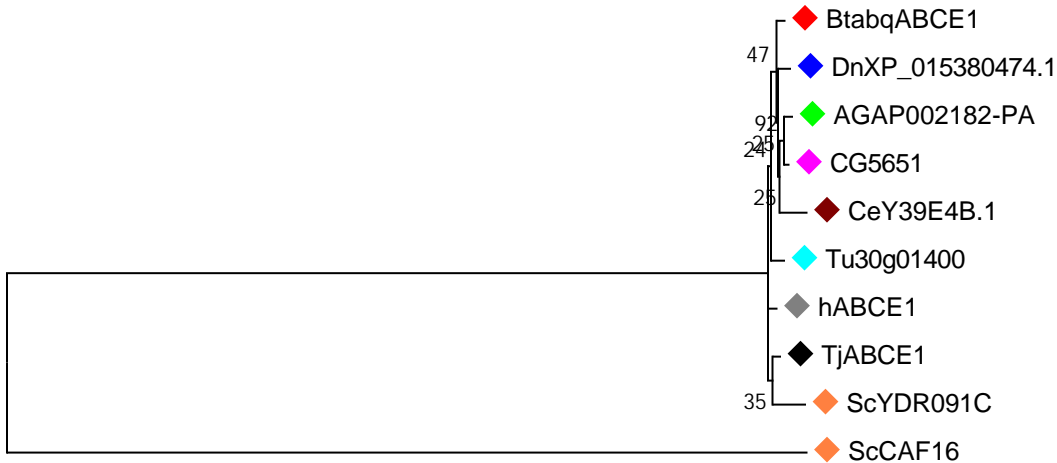

Supplement: Supplementary file 6 — Phylogenetic relationship of Bemisia tabaci ABCE subfamily with other organisms. See Figure S2 legend for details. (PDF 37 kb) [file 12864_2017_3706_MOESM6_ESM.pdf]

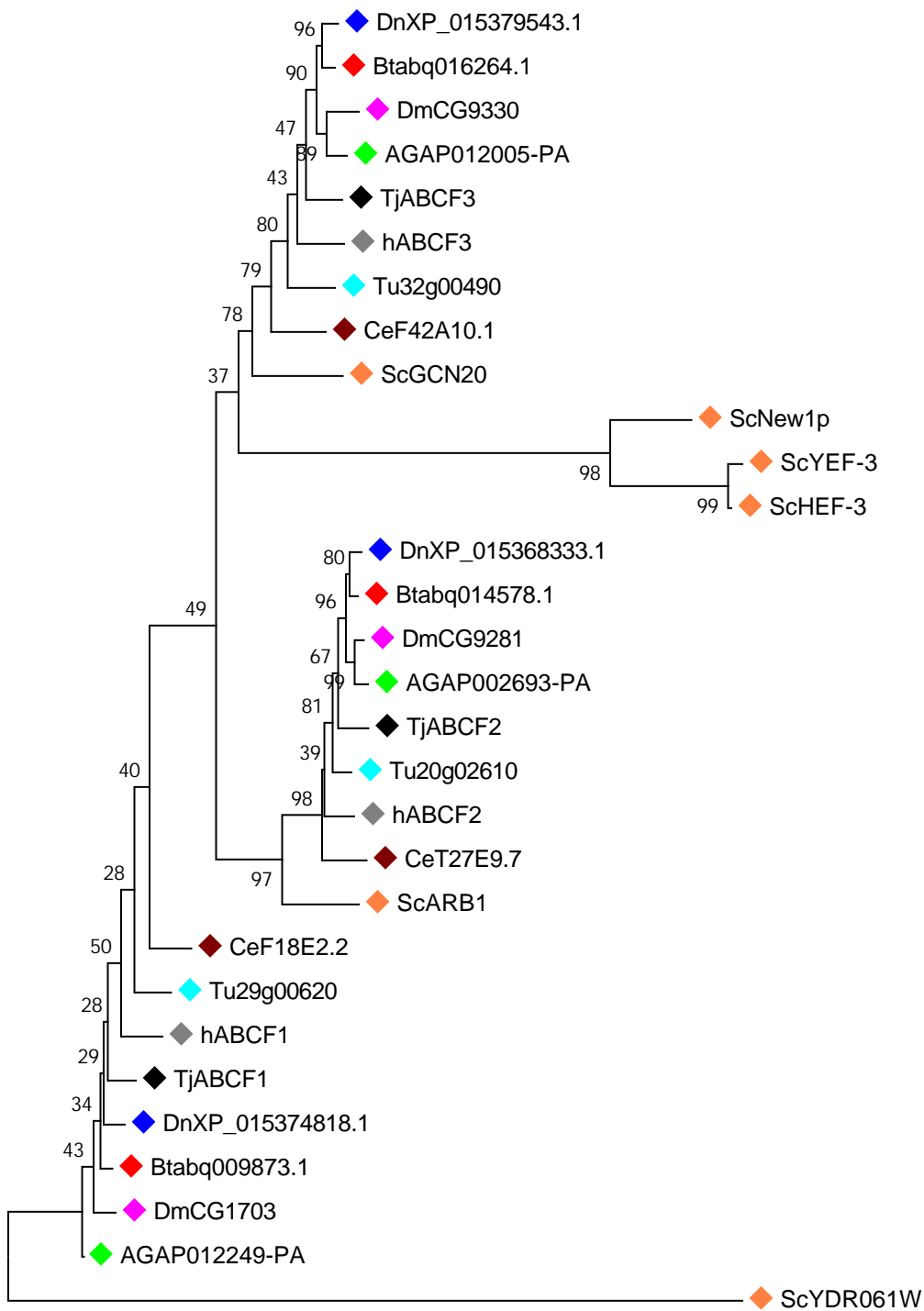

0.5

Supplement: Supplementary file 7 — Phylogenetic relationship of Bemisia tabaci ABCF subfamily with other organisms. See Figure S2 legend for details. (PDF 89 kb) [file 12864_2017_3706_MOESM7_ESM.pdf]

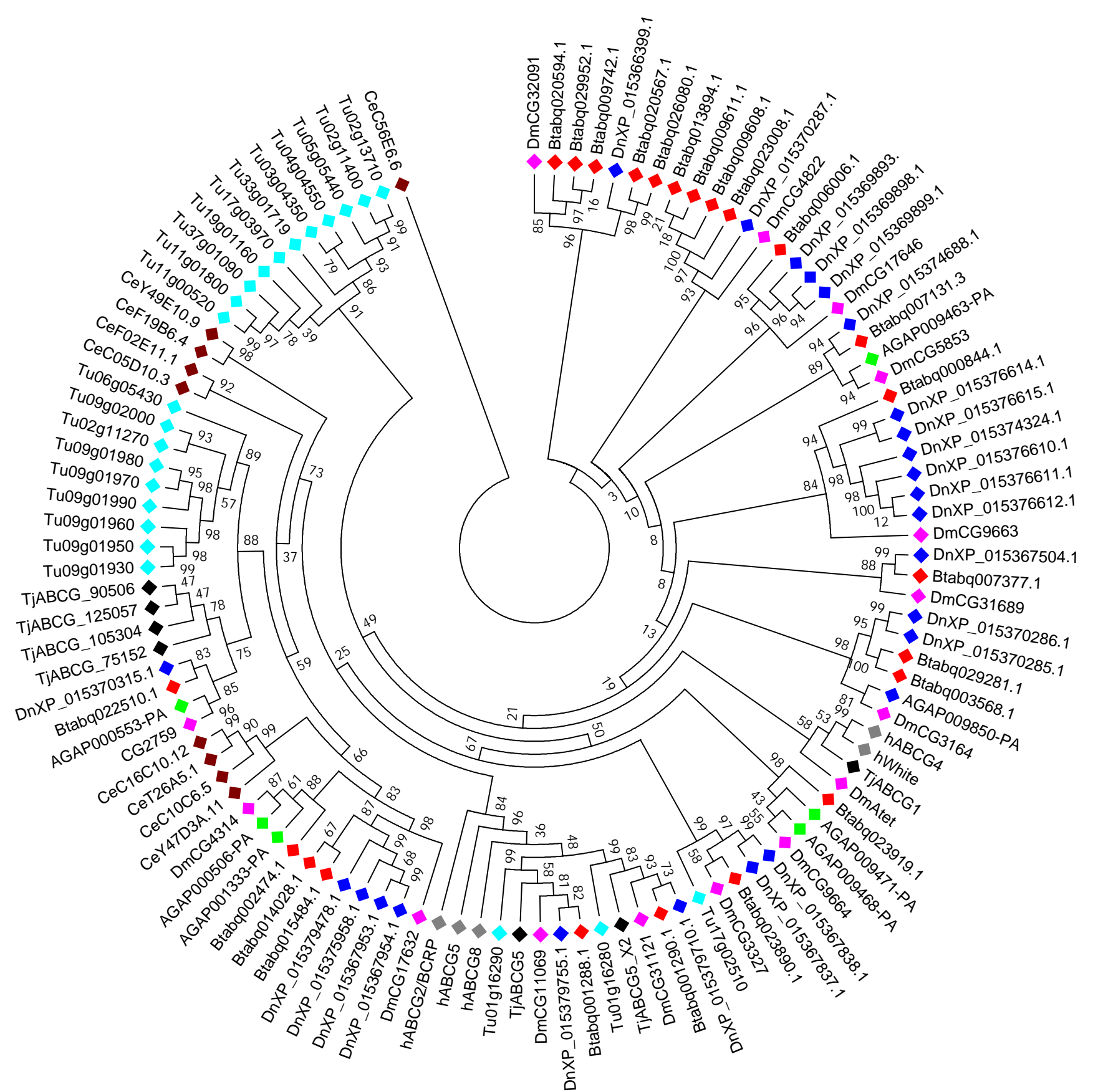

Supplement: Supplementary file 8 — Phylogenetic relationship of Bemisia tabaci ABCG subfamily with other organisms. See Figure S2 legend for details. (PDF 16 kb) [file 12864_2017_3706_MOESM8_ESM.pdf]

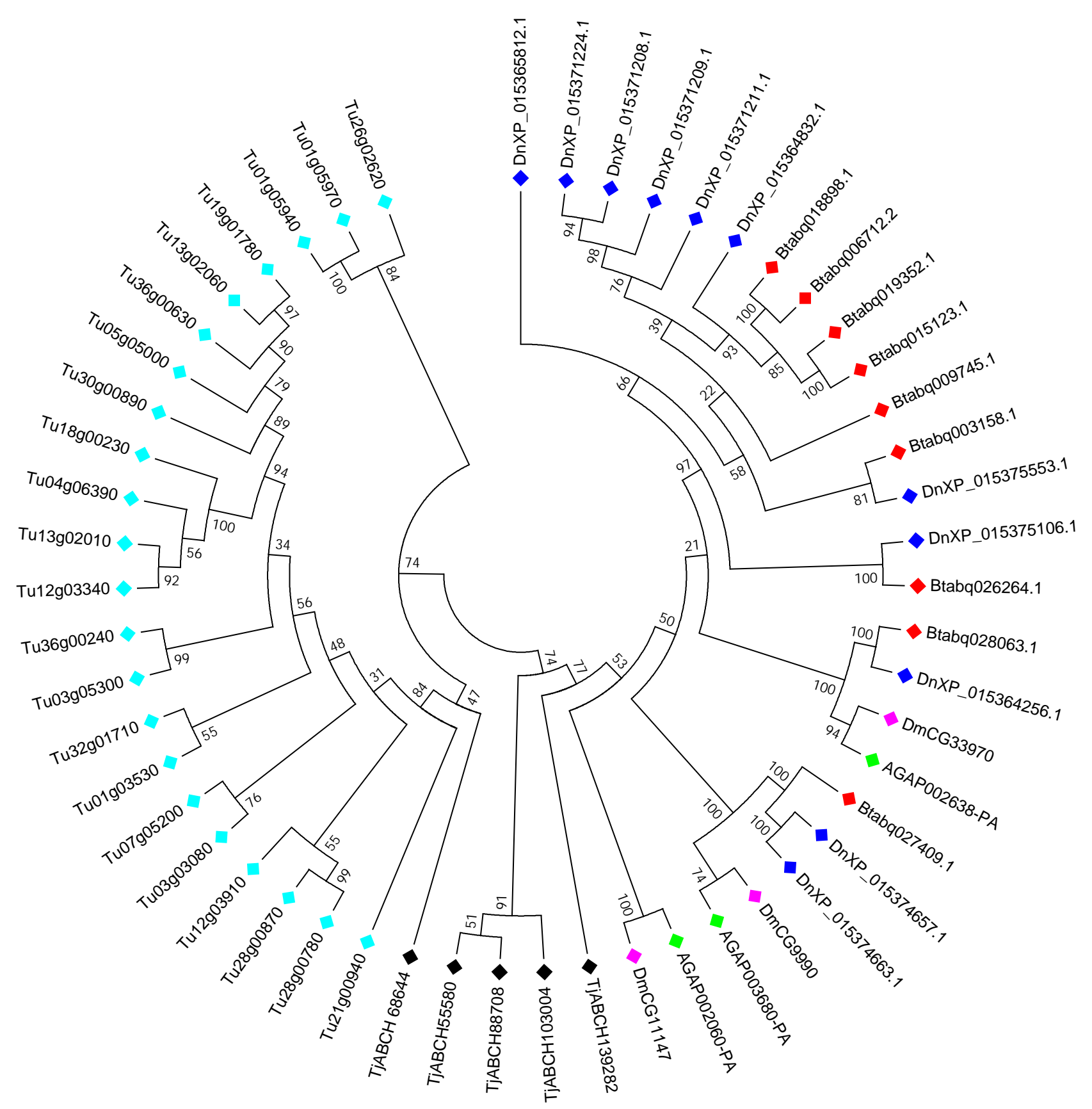

Supplement: Supplementary file 9 — Phylogenetic relationship of Bemisia tabaci ABCH subfamily with other organisms. See Figure S2 legend for details. (PDF 134 kb) [file 12864_2017_3706_MOESM9_ESM.pdf]
