# Supplementary material for: Genome-wide analysis of ATP-binding cassette (ABC) transporters in the sweetpotato whitefly, Bemisia tabaci
Source: BMC Genomics. 2017 Apr 26;18:330. doi: 10.1186/s12864-017-3706-6 (PMC5405539; doi:10.1186/s12864-017-3706-6)
Supplement: Supplementary file 10 — RPKM values of B. tabaci Q ABC transporters at different developmental stages (DOCX 13 kb) [file 12864_2017_3706_MOESM10_ESM.docx]

**Table S1. RPKM values of *B. tabaci* Q ABC transporters at different developmental stages.**

| ID | Subfamily | Egg | N1-2 | N3 | N4 | F | M |
| --- | --- | --- | --- | --- | --- | --- | --- |
| Btabq017043.1 | A | 8.659279725 | 9.454623 | 9.056951 | 9.056951 | 9.189508 | 9.101137 |
| Btabq003223.3 | A | 8.60740308 | 8.941244 | 8.774324 | 8.774324 | 8.829964 | 8.79287 |
| Btabq003232.1 | A | 13.6934344 | 12.64233 | 13.16788 | 13.16788 | 12.9927 | 13.10949 |
| Btabq009375.1 | A | 8.603020633 | 10.7191 | 9.661061 | 9.661061 | 10.01374 | 9.778621 |
| Btabq009376.1 | A | 8.935526064 | 11.59016 | 10.26284 | 10.26284 | 10.70528 | 10.41032 |
| Btabq022409.1 | A | 8.624313467 | 11.3324 | 9.978357 | 9.978357 | 10.4297 | 10.12881 |
| Btabq022410.1 | A | 8.268127724 | 10.82387 | 9.546 | 9.546 | 9.971957 | 9.687986 |
| Btabq008198.1 | A | 12.27090856 | 11.76366 | 12.01729 | 12.01729 | 11.93274 | 11.9891 |
| Btabq001304.1 | B | 12.17308069 | 12.16108 | 12.16708 | 12.16708 | 12.16508 | 12.16641 |
| Btabq028971.1 | B | 13.32427716 | 12.56846 | 12.94637 | 12.94637 | 12.8204 | 12.90438 |
| Btabq013065.1 | B | 11.13573147 | 10.9542 | 11.04496 | 11.04496 | 11.01471 | 11.03488 |
| Btabq000311.1 | C | 11.53948658 | 11.04931 | 11.2944 | 11.2944 | 11.2127 | 11.26717 |
| Btabq019529.2 | C | 12.26922935 | 12.29615 | 12.28269 | 12.28269 | 12.28718 | 12.28419 |
| Btabq026695.3 | C | 11.83765642 | 12.62184 | 12.22975 | 12.22975 | 12.36045 | 12.27332 |
| Btabq008004.1 | C | 12.6031933 | 12.39988 | 12.50154 | 12.50154 | 12.46765 | 12.49024 |
| Btabq004618.1 | C | 11.96040757 | 11.2497 | 11.60506 | 11.60506 | 11.48661 | 11.56557 |
| Btabq003933.1 | C | 10.66004069 | 10.09601 | 10.37802 | 10.37802 | 10.28402 | 10.34669 |
| Btabq026746.1 | D | 13.72117177 | 14.08958 | 13.90538 | 13.90538 | 13.96678 | 13.92584 |
| Btabq017051.1 | D | 11.73711919 | 10.78043 | 11.25877 | 11.25877 | 11.09933 | 11.20563 |
| BtabqABCE1 | E | 14.33964718 | 14.09546 | 14.21755 | 14.21755 | 14.17686 | 14.20399 |
| Btabq009873.1 | F | 14.49949691 | 13.95074 | 14.22512 | 14.22512 | 14.13366 | 14.19463 |
| Btabq014578.1 | F | 16.51121548 | 15.98531 | 16.24827 | 16.24827 | 16.16062 | 16.21905 |
| Btabq016264.1 | F | 13.384819 | 12.69492 | 13.03987 | 13.03987 | 12.92489 | 13.00154 |
| Btabq003568.1 | G | 14.358255 | 14.70579 | 14.53202 | 14.53202 | 14.58994 | 14.55133 |
| Btabq006006.1 | G | 13.00473047 | 12.98017 | 12.99245 | 12.99245 | 12.98836 | 12.99108 |
| Btabq007377.1 | G | 12.47211503 | 13.00167 | 12.73689 | 12.73689 | 12.82515 | 12.76631 |
| Btabq020594.1 | G | 11.72162787 | 11.91084 | 11.81624 | 11.81624 | 11.84777 | 11.82675 |
| Btabq029952.1 | G | 11.72873133 | 11.99819 | 11.86346 | 11.86346 | 11.90837 | 11.87843 |
| Btabq009742.1 | G | 11.93651788 | 12.46056 | 12.19854 | 12.19854 | 12.28588 | 12.22765 |
| Btabq029281.1 | G | 14.99421015 | 15.34917 | 15.17169 | 15.17169 | 15.23085 | 15.19141 |
| Btabq007131.3 | G | 13.52646526 | 12.92025 | 13.22336 | 13.22336 | 13.12232 | 13.18968 |
| Btabq023008.1 | G | 10.73894789 | 11.66859 | 11.20377 | 11.20377 | 11.35871 | 11.25542 |
| Btabq009608.1 | G | 11.19986758 | 12.27542 | 11.73764 | 11.73764 | 11.9169 | 11.7974 |
| Btabq009611.1 | G | 11.05739952 | 12.12203 | 11.58971 | 11.58971 | 11.76715 | 11.64886 |
| Btabq023919.1 | G | 11.00473116 | 11.29334 | 11.14903 | 11.14903 | 11.19713 | 11.16507 |
| Btabq013894.1 | G | 10.75180392 | 11.77639 | 11.2641 | 11.2641 | 11.43486 | 11.32102 |
| Btabq000844.1 | G | 13.29730841 | 12.63869 | 12.968 | 12.968 | 12.85823 | 12.93141 |
| Btabq026080.1 | G | 10.67746419 | 12.57116 | 11.62431 | 11.62431 | 11.93992 | 11.72951 |
| Btabq020567.1 | G | 10.86158619 | 12.75142 | 11.8065 | 11.8065 | 12.12148 | 11.91149 |
| Btabq001288.1 | G | 14.36361892 | 14.14798 | 14.2558 | 14.2558 | 14.21986 | 14.24382 |
| Btabq001290.1 | G | 14.5265324 | 13.98953 | 14.25803 | 14.25803 | 14.16853 | 14.2282 |
| Btabq023890.1 | G | 13.0261086 | 12.50365 | 12.76488 | 12.76488 | 12.6778 | 12.73585 |
| Btabq014028.1 | G | 11.87165641 | 12.28571 | 12.07869 | 12.07869 | 12.1477 | 12.10169 |
| Btabq015484.1 | G | 13.25132804 | 12.38438 | 12.81785 | 12.81785 | 12.67336 | 12.76969 |
| Btabq002474.1 | G | 9.199614178 | 6.920484 | 8.060049 | 8.060049 | 7.680194 | 7.933431 |
| Btabq022510.1 | G | 13.33770275 | 12.82136 | 13.07953 | 13.07953 | 12.99347 | 13.05085 |
| Btabq015123.1 | H | 11.05884933 | 12.86433 | 11.96159 | 11.96159 | 12.2625 | 12.06189 |
| Btabq003158.1 | H | 11.83695064 | 13.09408 | 12.46552 | 12.46552 | 12.67504 | 12.53536 |
| Btabq006712.2 | H | 9.199375514 | 11.18189 | 10.19063 | 10.19063 | 10.52105 | 10.30077 |
| Btabq018898.1 | H | 8.028763549 | 13.39669 | 10.71272 | 10.71272 | 11.60738 | 11.01094 |
| Btabq019352.1 | H | 11.3341853 | 13.1214 | 12.22779 | 12.22779 | 12.52566 | 12.32708 |
| Btabq026264.1 | H | 12.63255481 | 13.45017 | 13.04136 | 13.04136 | 13.17763 | 13.08678 |
| Btabq028063.1 | H | 11.23467834 | 12.4336 | 11.83414 | 11.83414 | 12.03396 | 11.90074 |
| Btabq009745.1 | H | 12.15286986 | 13.77964 | 12.96625 | 12.96625 | 13.23738 | 13.05663 |
| Btabq027409.1 | H | 14.06275423 | 15.12484 | 14.5938 | 14.5938 | 14.77081 | 14.6528 |

E, egg; N1-2, 1st- and 2nd-instar nymphs; N3, 3rd-instar nymph; N4, 4th-instar nymph; F, adult female; M, adult male. Values are the normalized data from three biological replicates.
