# Supplementary material for: Genome-wide analysis of ATP-binding cassette (ABC) transporters in the sweetpotato whitefly, Bemisia tabaci
Source: BMC Genomics. 2017 Apr 26;18:330. doi: 10.1186/s12864-017-3706-6 (PMC5405539; doi:10.1186/s12864-017-3706-6)
Supplement: Supplementary file 11 — Fold-change of gene expression following B. tabaci Q exposure to imidacloprid (DOCX 8 kb) [file 12864_2017_3706_MOESM11_ESM.docx]

**Table S4. Fold-change of gene expression following *B. tabaci* Q exposure to imidacloprid**

| **ID** | **Subfamily** | **FC**  **(IM25)** | **log_2_FC (IM25)** | **Padj**  **(IM25)** | **FC**  **(IM50)** | **log_2_FC (IM50)** | **Padj (IM50)** | **FC**  **(IM100)** | **log_2_FC (IM100)** | **Padj (IM100)** |
| --- | --- | --- | --- | --- | --- | --- | --- | --- | --- | --- |
| Btabq003223.3 | A | 0.943294781 | -0.084219409 | 1 | 0.926652397 | -0.109899833 | 1 | 1.047936125 | 0.067550783 | 1 |
| Btabq003232.1 | A | 0.781532735 | -0.355621792 | 1 | 0.847579516 | -0.238579373 | 1 | 1.074144349 | 0.103187882 | 1 |
| Btabq009375.1 | A | 1.125511792 | 0.170581172 | 1 | 1.677093411 | 0.745963046 | 1 | 1.085051377 | 0.117763355 | 1 |
| Btabq017043.1 | A | 0.870928034 | -0.199374584 | 1 | 0.941700478 | -0.086659833 | 1 | 1.090529431 | 0.125028705 | 1 |
| Btabq009376.1 | A | 1.050976862 | 0.071730907 | 1 | 1.273609925 | 0.348923484 | 1 | 1.415981239 | 0.501802151 | 0.227726 |
| Btabq022409.1 | A | 1.057275709 | 0.080351641 | 1 | 1.317293186 | 0.397576478 | 1 | 1.4212142 | 0.507124009 | 0.280321 |
| Btabq022410.1 | A | 1.461029944 | 0.546985747 | 1 | 1.887177234 | 0.91622992 | 1 | 1.550670419 | 0.632892087 | 0.582305 |
| Btabq008198.1 | A | 1.974186216 | 0.981258079 | 1 | 1.22311487 | 0.290559902 | 1 | 3.159542221 | 1.659715544 | 0.002176 |
| Btabq001304.1 | B | 0.849679478 | -0.235009374 | 1 | 1.013361314 | 0.019148659 | 1 | 1.089881782 | 0.124171657 | 1 |
| Btabq013065.1 | B | 1.044488441 | 0.062796527 | 1 | 1.099729152 | 0.137148251 | 1 | 0.993771434 | -0.009014023 | 1 |
| Btabq028971.1 | B | 1.044094283 | 0.062251996 | 1 | 0.966129169 | -0.049712009 | 1 | 1.068707309 | 0.095866791 | 1 |
| Btabq004618.1 | C | 1.031233442 | 0.044370955 | 1 | 1.095834798 | 0.132030323 | 1 | 1.069616374 | 0.097093456 | 1 |
| Btabq008004.1 | C | 1.016686065 | 0.023874268 | 1 | 1.046659016 | 0.065791512 | 1 | 1.173124533 | 0.230356171 | 0.772023 |
| Btabq026695.3 | C | 1.319199749 | 0.399663029 | 1 | 1.568768025 | 0.649632036 | 1 | 1.788839793 | 0.839024187 | 0.031496 |
| Btabq000311.1 | C | 0.882739086 | -0.179941017 | 1 | 1.249666688 | 0.321543349 | 1 | 1.011580238 | 0.016610758 | 1 |
| Btabq019529.2 | C | 0.935480532 | -0.096220464 | 1 | 1.102878563 | 0.141273946 | 1 | 1.445689587 | 0.531757815 | 0.314397 |
| Btabq003933.1 | C | 0.965264166 | -0.051004273 | 1 | 0.938440435 | -0.091662918 | 1 | 0.78688206 | -0.345780678 | 0.758665 |
| Btabq017051.1 | D | 0.842347701 | -0.247512228 | 1 | 0.956719853 | -0.063831559 | 1 | 0.756958716 | -0.401713477 | 0.644241 |
| Btabq026746.1 | D | 1.17436163 | 0.231876737 | 1 | 0.854798315 | -0.226344031 | 1 | 1.52889047 | 0.612485055 | 0.193829 |
| BtabqABCE1 | E | 0.91978384 | -0.120633245 | 1 | 0.920504612 | -0.119503146 | 1 | 1.151886333 | 0.203998361 | 0.781145 |
| Btabq009873.1 | F | 0.926030745 | -0.110868001 | 1 | 0.865632656 | -0.208173169 | 1 | 0.933648303 | -0.099048894 | 1 |
| Btabq014578.1 | F | 1.004904953 | 0.007059053 | 1 | 0.936981623 | -0.093907342 | 1 | 0.866013725 | -0.207538205 | 0.682927 |
| Btabq016264.1 | F | 0.98282186 | -0.024998148 | 1 | 0.839780226 | -0.251916277 | 1 | 0.963229464 | -0.054048572 | 1 |
| Btabq000844.1 | G | 0.991677812 | -0.012056618 | 1 | 0.834811345 | -0.260477888 | 1 | 0.866872628 | -0.206108065 | 0.954814 |
| Btabq001288.1 | G | 1.10988697 | 0.150412762 | 1 | 1.02746371 | 0.039087439 | 1 | 1.643508866 | 0.716779239 | 0.236721 |
| Btabq003568.1 | G | 1.234451711 | 0.303870403 | 1 | 0.839213961 | -0.252889416 | 1 | 2.095271184 | 1.067136979 | 0.000288 |
| Btabq006006.1 | G | 0.915238791 | -0.127779895 | 1 | 0.999500022 | -0.000721497 | 1 | 1.254358477 | 0.326949707 | 0.725081 |
| Btabq007131.3 | G | 1.67398074 | 0.743282929 | 1 | 0.762905517 | -0.390423699 | 1 | 3.46559457 | 1.793102888 | 5.20E-05 |
| Btabq007377.1 | G | 1.978610713 | 0.984487794 | 1 | 1.257567411 | 0.330635737 | 1 | 2.333228462 | 1.222327578 | 0.131568 |
| Btabq009608.1 | G | 0.824443411 | -0.278507624 | 1 | 0.969537285 | -0.044631714 | 1 | 2.087404282 | 1.061710044 | 0.155944 |
| Btabq009611.1 | G | 0.742953788 | -0.428655617 | 1 | 0.880148235 | -0.184181572 | 1 | 1.864941863 | 0.899130658 | 0.322169 |
| Btabq013894.1 | G | 0.928753943 | -0.106631665 | 1 | 0.86806702 | -0.204121663 | 1 | 1.966632178 | 0.975727153 | 0.357209 |
| Btabq014028.1 | G | 0.998676177 | -0.001911138 | 1 | 0.871565233 | -0.198319446 | 1 | 1.059588992 | 0.083504761 | 1 |
| Btabq015484.1 | G | 1.01889393 | 0.027003871 | 1 | 0.970993304 | -0.042466749 | 1 | 0.976644041 | -0.034095258 | 1 |
| Btabq020567.1 | G | 0.737362646 | -0.439553762 | 1 | 1.072155776 | 0.100514534 | 1 | 1.581454021 | 0.661251611 | 0.970897 |
| Btabq020594.1 | G | 1.711321261 | 0.775110618 | 1 | 1.098728852 | 0.135835397 | 1 | 2.821478412 | 1.496451311 | 0.038455 |
| Btabq023008.1 | G | 0.850717257 | -0.233248374 | 1 | 0.883754551 | -0.178282355 | 1 | 1.938884981 | 0.955227222 | 0.365455 |
| Btabq023890.1 | G | 0.626455315 | -0.67471649 | 1 | 0.980188756 | -0.028868497 | 1 | 0.906471994 | -0.141665648 | 1 |
| Btabq023919.1 | G | 0.989909158 | -0.014631957 | 1 | 0.852508428 | -0.230213998 | 1 | 1.292226978 | 0.3698595 | 0.890444 |
| Btabq029281.1 | G | 1.296322054 | 0.374424181 | 1 | 0.913994272 | -0.129742971 | 1 | 2.457516343 | 1.297201011 | 1.29E-07 |
| Btabq029952.1 | G | 1.086970065 | 0.12031221 | 1 | 1.016825355 | 0.02407191 | 1 | 2.220227219 | 1.15070733 | 0.160081 |
| Btabq022510.1 | G | 0.923132174 | -0.115390868 | 1 | 0.960259895 | -0.05850317 | 1 | 0.845992404 | -0.241283385 | 0.573845 |
| Btabq001290.1 | G | 1.002387628 | 0.003440513 | 1 | 1.030871738 | 0.043864843 | 1 | 0.942251874 | -0.085815336 | 1 |
| Btabq002474.1 | G | 1.167013671 | 0.222821461 | 1 | 0.600096675 | -0.736733158 | 1 | 1.37844365 | 0.463040292 | 1 |
| Btabq009742.1 | G | 1.677655341 | 0.746446358 | 1 | 1.514425884 | 0.598770974 | 1 | 3.390799162 | 1.761625336 | 0.15872 |
| Btabq026080.1 | G | 0.518176185 | -0.948485382 | 1 | 0.840359016 | -0.250922291 | 1 | 0.885102384 | -0.176083747 | 1 |
| Btabq005174.1 | G | 0.579677084 | -0.786678641 | 1 | 0.492515529 | -1.021758882 | 1 | 2.31315931 | 1.209864629 | 0.683468 |
| Btabq015123.1 | H | 0.961052871 | -0.057312294 | 1 | 0.830384283 | -0.268148958 | 1 | 0.900618363 | -0.151012201 | 0.948248 |
| Btabq003158.1 | H | 0.877058579 | -0.189254891 | 1 | 0.840148889 | -0.251283073 | 1 | 0.77365998 | -0.370228447 | 0.584766 |
| Btabq006712.2 | H | 1.339234142 | 0.421408213 | 1 | 1.110977189 | 0.151829196 | 1 | 1.640048131 | 0.713738155 | 0.464811 |
| Btabq018898.1 | H | 0.708191165 | -0.497789249 | 1 | 0.784336968 | -0.350454494 | 1 | 0.640511068 | -0.642704595 | 0.845292 |
| Btabq019352.1 | H | 0.960517084 | -0.05811682 | 1 | 0.826924628 | -0.274172257 | 1 | 0.89628188 | -0.157975564 | 0.911687 |
| Btabq026264.1 | H | 0.90236808 | -0.14821206 | 1 | 0.626420323 | -0.674797077 | 1 | 1.423573749 | 0.509517235 | 0.804018 |
| Btabq028063.1 | H | 0.770400631 | -0.376319211 | 1 | 0.47003493 | -1.089160122 | 1 | 1.838168654 | 0.878269141 | 0.780775 |
| Btabq027409.1 | H | 0.926130334 | -0.110712857 | 1 | 0.848450037 | -0.23709839 | 1 | 1.464875795 | 0.550778346 | 0.35824 |
| Btabq009745.1 | H | 1.433355352 | 0.519396321 | 1 | 0.806388587 | -0.310452875 | 1 | 1.360239086 | 0.006085764 | 0.08593 |

IM25, IM50, or IM100 refers to *B. tabaci* Q treated with imidacloprid at 25, 50, or 100 mg/L.

Padj refers to the adjusted P value.
