# Supplementary material for: Genome-wide analysis of ATP-binding cassette (ABC) transporters in the sweetpotato whitefly, Bemisia tabaci
Source: BMC Genomics. 2017 Apr 26;18:330. doi: 10.1186/s12864-017-3706-6 (PMC5405539; doi:10.1186/s12864-017-3706-6)
Supplement: Supplementary file 12 — Models used for phylogenetic analysis (DOCX 15 kb) [file 12864_2017_3706_MOESM12_ESM.docx]

**Table S5 Models used for phylogenetic analysis**

|  | **ABC transporters** | **Subfamily** | | | | | | | |
| --- | --- | --- | --- | --- | --- | --- | --- | --- | --- |
|  |  | **A** | **B** | **C** | **D** | **E** | **F** | **G** | **H** |
| **Model** | WAG+G | WAG+G+F | WAG+G | rtREV+G+I+F | rtREV+G+F | WAG+G | rtREV+G+F | WAG+G+F | WAG+G+I+F |
