# Supplementary material for: Genome-wide analysis of ATP-binding cassette (ABC) transporters in the sweetpotato whitefly, Bemisia tabaci
Source: BMC Genomics. 2017 Apr 26;18:330. doi: 10.1186/s12864-017-3706-6 (PMC5405539; doi:10.1186/s12864-017-3706-6)
Supplement: Supplementary file 13 — Selected B. tabaci Q ABC transporters for the RT-qPCR validation study (DOCX 143 kb) [file 12864_2017_3706_MOESM13_ESM.docx]

**Table S2. Selected *B. tabaci* Q ABC transporters for the RT-qPCR validation study.**

| **Expression level**^*^ | **Subfamily** | | | | | | | |
| --- | --- | --- | --- | --- | --- | --- | --- | --- |
|  | **A** | **B** | **C** | **D** | **E** | **F** | **G** | **H** |
| >=13 | Btabq003232.1 | Btabq028971.1 |  | Btabq026746.1 | BtabqABCE1 | Btabq009873.1  Btabq014578.1 | Btabq006006.1  Btabq029281.1  Btabq001288.1 | Btabq026264.1  Btabq027409.1 |
| 11--13 | Btabq008198.1 | Btabq001304.1 | Btabq019529.2 | Btabq017051.1 |  |  | Btabq014028.1  Btabq015484.1 | Btabq003158.1  Btabq028063.1 |
| <=11 | Btabq017043.1 | Btabq013065.1 | Btabq003933.1 |  |  |  | Btabq002474.1 | Btabq006712.2 |

^1^

^*^ Log_2_(RPKM+1) values based on RNA-seq analysis.
