# Supplementary material for: Genome-wide analysis of ATP-binding cassette (ABC) transporters in the sweetpotato whitefly, Bemisia tabaci
Source: BMC Genomics. 2017 Apr 26;18:330. doi: 10.1186/s12864-017-3706-6 (PMC5405539; doi:10.1186/s12864-017-3706-6)
Supplement: Supplementary file 14 — Primers used for the RT-qPCR analysis (DOCX 89 kb) [file 12864_2017_3706_MOESM14_ESM.docx]

**Table S3. Primers used for the RT-qPCR analysis**

| **Gene** | **Primer (5’ -3’)** | **E** |
| --- | --- | --- |
| Btabq003232.1-F | CTGGTGCGTTTTGTGCCC | 110% |
| Btabq003232.1-R | CAATGCGTCAATAGCCAAC |  |
| Btabq008198.1-F | CCGATAAGCCTTGCCACAGAC | 103% |
| Btabq008198.1-R | TGAGAAGATTATGAAGAGGAGACCC |  |
| Btabq017043.1-F | GCGGACGAGTCGGAGATG | 96% |
| Btabq017043.1-R | CGGTGTTGAACTGGTTAGAGGC |  |
| Btabq028971.1-F | GCTTCCTGGGGTCGGTCT | 105% |
| Btabq028971.1-R | TGGAGCTTGCACTTTACTCCTAAT |  |
| Btabq001304.1-F | GCCCTCTTCGTTACACGCTA | 100% |
| Btabq001304.1-R | CTATGCCATGTTGACTGATTTGA |  |
| Btabq013065.1-F | AAGCCGACCTCCCAGCAC | 110% |
| Btabq013065.1-R | CATCACATTGACCGCCACA |  |
| Btabq019529.2-F | CCAGAACTAGCGGCGTCCTT | 100% |
| Btabq019529.2-R | AGATTGCTTTGCTACTGTTTTCCT |  |
| Btabq003933.1-F | GGCAGTTTGTCCCTTTTCGG | 104% |
| Btabq003933.1-R | TGTTTTGTCATCTTCATTACATTCG |  |
| Btabq026746.1-F | GGACGGGCGGGTTGAA | 90% |
| Btabq026746.1-R | GTTTGGTGAGGTGCGAGTAGAG |  |
| Btabq017051.1-F | TGCTTTCTATCAAGGGAACAATC | 106% |
| Btabq017051.1-R | AACTTGCGTAAATGCTGGGTAA |  |
| BtabqABCE1-F | ACCCGCCTGATTGGACG | 110% |
| BtabqABCE1-R | CCTTTGACAGCCTTTGGTATTT |  |
| Btabq009873.1-F | CGACGAAGACAAACCCACAG | 91% |
| Btabq009873.1-R | TAGGACCTACGATAGCAACACGA |  |
| Btabq014578.1-F | ACATGATGAAATCGTTCCCAGAT | 107% |
| Btabq014578.1-R | TGCCTTCCAGTAAGCCCGTA |  |
| Btabq006006.1-F | GAAACTAAAGTCAACGGCGAAAC | 94% |
| Btabq006006.1-R | GAGTAGGAGACATCCCTGAACGA |  |
| Btabq029281.1-F | CGTTGAGTCGTGGTGGTCG | 94% |
| Btabq029281.1-R | AGTAAGAGGCTGGGTTGTGGTA |  |
| Btabq001288.1-F | CTACTCCTTGCCACTTTCACTCA | 91% |
| Btabq001288.1-R | CACAAACGAACTCTTTACAACCAT |  |
| Btabq014028.1-F | TTTACATGGCCTGGATCAAATAC | 93% |
| Btabq014028.1-R | CGACACTTTTAGAGCACGGAATA |  |
| Btabq015484.1-F | GGTTTCCTGGGCTTCTG | 101% |
| Btabq015484.1-R | ACACCAAGGCAGCGAAT |  |
| Btabq002474.1-F | TACACGGACTCACTGGATG | 97% |
| Btabq002474.1-R | CCTTGAACCGACTGGATA |  |
| Btabq026264.1-F | TCCAATCGACATTGATTTAAGCG | 96% |
| Btabq026264.1-R | GGGTTGTTGTTTCCATAGATAGCAT |  |
| Btabq027409.1-F | GAAGTTGGGTTGCAGGAGTTAC | 100% |
| Btabq027409.1-R | CCGAAGCACATGCCACATA |  |
| Btabq003158.1-F | TACTCATCTCCCGCCATTCTT | 108% |
| Btabq003158.1-R | CGTTTTCACTCCTGCCACTAA |  |
| Btabq028063.1-F | CAGAGGCGGAAGCCATAGG | 108% |
| Btabq028063.1-R | GCGTCCATTCGCTCCTTGA |  |
| Btabq006712.2-F | TGGTTGGACTTATGGGTGCG | 110% |
| Btabq006712.2-R | TGCATGGTCGTCAGGAAGG |  |

E, indicates PCR efficiency.
